# Modular assembly of transposable element arrays by microsatellite targeting in the guayule and rice genomes

**DOI:** 10.1186/s12864-018-4653-6

**Published:** 2018-04-19

**Authors:** José A. Valdes Franco, Yi Wang, Naxin Huo, Grisel Ponciano, Howard A. Colvin, Colleen M. McMahan, Yong Q. Gu, William R. Belknap

**Affiliations:** 1 Universidad Autónoma de Nuevo León, Monterrey, NL Mexico; 2 USDA-Agricultural Research Service, Western Regional Research Center, Albany, CA USA; 3 Cooper Tire & Rubber Company, Findlay, OH USA; 4 Present Address: Plant Breeding and Genetics Section, School of Integrative Plant Science, Cornell University, Ithaca, NY USA

**Keywords:** Natural rubber, Genome, Assembly, Annotation, Class II transposable element, Non-autonomous, Transposon

## Abstract

**Background:**

Guayule (*Parthenium argentatum* A. Gray) is a rubber-producing desert shrub native to Mexico and the United States. Guayule represents an alternative to *Hevea brasiliensis* as a source for commercial natural rubber. The efficient application of modern molecular/genetic tools to guayule improvement requires characterization of its genome.

**Results:**

The 1.6 Gb guayule genome was sequenced, assembled and annotated. The final 1.5 Gb assembly, while fragmented (N_50_ = 22 kb), maps > 95% of the shotgun reads and is essentially complete. Approximately 40,000 transcribed, protein encoding genes were annotated on the assembly. Further characterization of this genome revealed 15 families of small, microsatellite-associated, transposable elements (TEs) with unexpected chromosomal distribution profiles. These *SaTar* (Satellite Targeted) elements, which are non-autonomous *Mu-*like elements (MULEs), were frequently observed in multimeric linear arrays of unrelated individual elements within which no individual element is interrupted by another. This uniformly non-nested TE multimer architecture has not been previously described in either eukaryotic or prokaryotic genomes. Five families of similarly distributed non-autonomous MULEs (microsatellite associated, modularly assembled) were characterized in the rice genome. Families of TEs with similar structures and distribution profiles were identified in sorghum and citrus.

**Conclusion:**

The sequencing and assembly of the guayule genome provides a foundation for application of current crop improvement technologies to this plant. In addition, characterization of this genome revealed *SaTar* elements with distribution profiles unique among TEs. *Satar* targeting appears based on an alternative MULE recombination mechanism with the potential to impact gene evolution.

**Electronic supplementary material:**

The online version of this article (10.1186/s12864-018-4653-6) contains supplementary material, which is available to authorized users.

## Background

Guayule, a species in the Compositae (Asteraceae) family, is a perennial shrub native to the Chihuahuan Desert of North America that represents a potential commercial source of natural rubber [1]. There have been the numerous unsuccessful past efforts to develop guayule as a crop [2], however, both a narrow germplasm pool and the innate genetic complexity of guayule limited past breeding improvement efforts [1]. The importance of guayule, and other plants, as alternatives to *Hevea brasiliensis* as natural rubber sources can be appreciated when the economics and supply of *Hevea* rubber are considered. Seventy percent of worldwide rubber production (12.3 million metric tons in 2015) is utilized in tire fabrication [3], and natural rubber represents one of the largest corporate purchases made by this industry. The natural rubber supply is subject to both considerable price volatility and a number of issues associated with security of supply. For example, the *Hevea* tree is susceptible to South American Leaf Blight (SALB) caused by the endemic fungus *Microcyclus ulei*. SALB terminated commercial *Hevea* cultivation in South America in the early 1900’s and remains a major threat to rubber production [4]. Thus, identification and development of alternative sources of natural rubber are important to the tire industry. The sequencing and annotation of the 1.6 Gb [5, 6] nuclear genome of a diploid guayule was undertaken to facilitate the application of current molecular and breeding tools to guayule improvement.

The genome annotation process, which follows sequence acquisition and assembly, involves identification of encoded gene and structural components, essentially converting a compiled DNA sequence into an information-rich tool with broad applicability. The identification/characterization of repeat sequences is a requisite first step for efficient and accurate genome annotation [7]. In most chromosomal domains, the repeated DNA sequences represent largely transposable elements (TEs) of two classes. The class I retrotransposons are mobilized through an RNA intermediate and make up large percentages of plant genomes [8]. The class II transposons mobilize through a DNA intermediates, and exist both in autonomous (TE encodes requisite transposase proteins) and non-autonomous (transposase function supplied in *trans*) forms [9]. Both TE types have non-random distribution profiles on plant chromosomes, with the class II TEs associated with gene-rich chromosomal regions [9]. The *Mu*-like element (MULE) superfamily of class II TEs represents a large and diverse set of autonomous and non-autonomous elements that can make up a significant percentage of plant genomes [10, 11]. The MULE TEs, particularly the Pack-MULEs, have been proposed as important mediators of plant gene evolution [12–15].

In the course of characterizing repeated sequences in initial guayule genome assemblies, a number of short, unrelated repetitive DNA sequences associated with microsatellites were identified. Microsatellites, or simple sequence repeats (SSRs), are regions of tandemly repeated short (1-6 bp) DNA repeats that are common features in genomes [16]. The guayule microsatellite-associated elements were identified as microsatellite-targeted non-autonomous MULE elements by both structural and sequence similarities to guayule autonomous MULEs. While specific microsatellite-associated class II TEs [17–19], including autonomous MULE elements [20, 21], have been identified in plants, the elements in guayule had unique and unexpected features.

## Methods

### Plant material

All genomic and transcriptome sequences were derived from a single diploid guayule plant, accession W6 429 developed from a selection obtained in a 1942 Durango, Mexico collection expedition [1, 5, 6].

### Sequence acquisition

DNA was prepared as described previously [22], polyA-RNA was prepared employing Qiagen RNeasy/QIAshredder protocols.

Illumina (300 bp paired-end) shotgun sequencing libraries were made using the Kapa Biosystems protocol: High-Throughput NGS LibraryPreparation Technical Guide for Illumina TM platforms (KR0427 - V1.12) and sequenced with MiSeq ® Reagent Kit v3 Reagents on an Illumina MiSeq. The Roche 454 sequencing libraries were prepared using the Rapid Library Preparation Manual for GSFLX+ and GS Junior + Series (May 2011) and sequenced with Roche 454 GSFLX +. The Illumina matepair libraries with insert sizes of 500, 700 and 1 kb were made following the Nextera® Mate Pair Library Illumina HiSeq 2500.

Transcriptome sequence was obtained from polyA-RNA libraries constructed following the Kapa Biosystems protocol: KAPA Stranded mRNA-Seq Kit for illumina® platform (KR0960 -v3.15) and sequenced on an Illumina HiSeq 2500 (10^8^ × 150 bp reads). Greater than 80% of the transcriptome reads mapped to the Meraculous assembly.

### Meraculous and CLC Genomics Workbench genome assemblies

Meraculous is a whole genome assembler for next generation sequencing data geared for large genomes [23]. The guayule genome was first assembled using the Mercaculous assembler with the Illumina reads (shotgun and Nextera). This assembly encompassed 1.5 Gb (N_50_ = 22Kb, 260 k Scaffolds) and mapped > 95% of the shotgun reads. CLC Genomics Workbench assembly (version 8.5.1, in conjunction with scaffolding using SSPACE version 2.0 [24]) employed the Illumina and Roche 454 reads and provided improved representation of simple sequence repeat domains. This assembly encompassed 0.9 GB (N_50_ = 28Kb, 59 k Scaffolds) and retained > 95% of the annotated genes.

### Transcriptome assembly

For annotation purposes, the transcriptome was assembled employing Cufflinks [25] and Trinity [26] assemblers, and further processed with PASA [27].

### Gene annotation

Genome repeat sequences were annotated with RepeatMasker. Approximately 40,000 protein-encoding, transcribed, genes were annotated (Trinity/MAKER/Cufflinks) [25, 26, 28] on the Meraculous assembly. Genome repeat sequences were annotated denovo with RepeatMasker (http://www.repeatmasker.org/webrepeatmaskerhelp.html). Additionally, Augustus gene prediction software (http://bioinf.uni-greifswald.de/augustus/) was employed to identify expressed genes on the 33 guayule scaffolds artificially assembled in Additional file 1. Expressed genes in this assembly were verified by BLASTp returns with E values less than e-15.

### Computational de novo identification of *SaTar* elements

Software designed to identify potential Satellite Targeted (*SaTar*) elements in any genome was employed. Entered scaffolds are scanned for repeated sequences (250-800 bp in length, ≥ 20% GC content) defined by TA microsatellite domains (≥ 12 bp in length). The program utilizes MISA to identify microsatellite domains (http://pgrc.ipk-gatersleben.de/misa/misa.html). Software and Users Guide available at: http://probes.pw.usda.gov/Guayule.

## Results

### Sequencing, assembly and annotation of the guayule genome

**Fig. 1:**
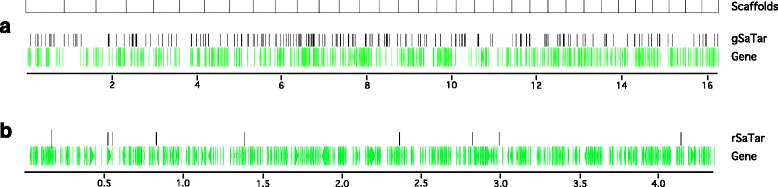
Expressed gene and SaTar element annotations on 1% of the guayule and rice genomes. **a** Artificial 16 Mb assembly of the 33 longest guayule scaffolds representing 1% of the guayule genome. **b** Rice (*Oryza sativa (japonica) v7_JGI* (34)) chromosome 1 sequence from 21.0–25.4 Mb. Expressed genes on the rice sequence annotated per the MSU Rice Genome Annotation Project Release 7 (35). The positions of annotated genes are indicated by green arrows. The guayule assembly contains 505 expressed genes and 215 *gSaTar* elements (69 of which are linked to other *gSaTars*). The rice genomic sequence contains 446 expressed genes and 10 *rSaTar* elements (two of which are linked)

The genes annotated from a single artificial 16 Mb assembly (approximately 1% of the genome) composed of the 33 longest Meraculous scaffolds is shown in Fig. 1a. The locations of the 505 annotated genes are indicated, a gene density consistent with predictions based on the overall genome (Table 1). The observed non-genic domains in this assembly are composed largely of retrotransposon and unclassified interspersed repetitive sequences (Table 1). However, no evidence for recent, widespread, amplification of specific Class I or Class II transposon/retrotransposon families was found in either the guayule assemblies or the collected reads. Syntenic analysis of the genes encoded on individual scaffolds was consistent with two rounds of genome-wide duplication in the last 40 MY [29].

**Table 1:** Genes and repetitive DNA on the 1.5 Gb Meraculous assemly

Mapped transcripts	Number		
CDS	268,632	cdhit uniques 25,740	
mRNA	61,170		
Full length ORF	44,750		
Refseq db	42,708		
Filter > 80%	39,347		
GO (swisprot 90%)	23,144		
Repetitive content (denovo)	Number	Content (bp)	Percentage
LTR	439,305	368,003,201	24.18%
LINEs	51,983	28,517,926	1.87%
SINEs	15,372	3,504,610	0.23%
DNA elements	196,517	51,599,240	3.39%
Unclassified	1,030,342	355,313,348	23.35%
Total interpersed repeats		806,938,325	53.02%
Small RNA	644	84,033	0.01%
Satellites	535	308,909	0.02%
Simple repeats	33,165	50,184,037	3.30%
Low complexity	44,121	2,189,640	0.14%

**Fig. 2:**
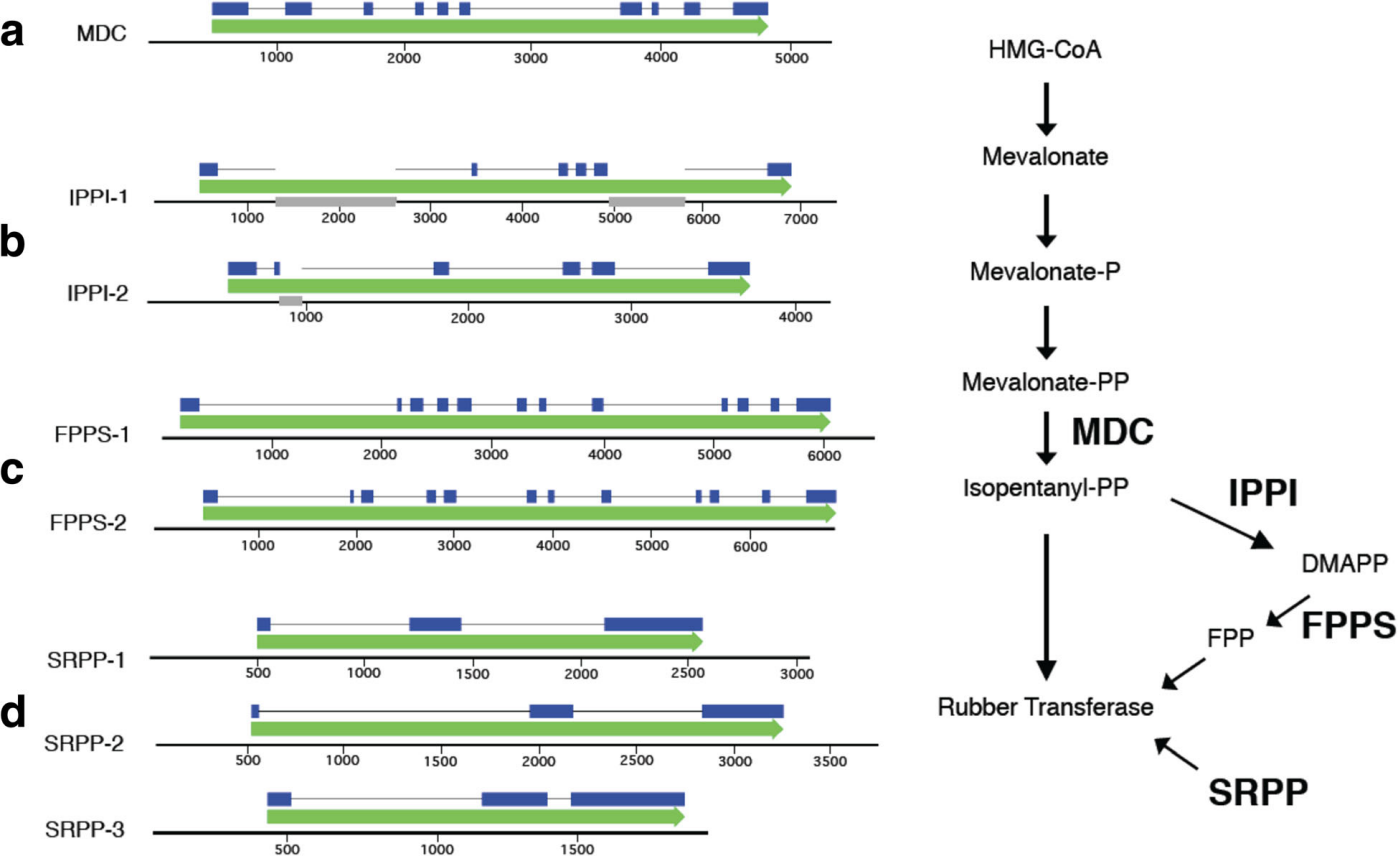
Structure of selected natural rubber biosynthetic genes in the guayule Meraculous genome assembly. Gene positions are indicated by green arrows, exons by blue boxes and unassembled domains by grey boxes. **a** Mevalonate Diphosphate Decarboxylase: 133969-128654 Scaffold126905. **b** Isopentenyl Diphosphate Isomerase: IPPI-1 34059-41432 Scaffold930862; IPPI-2 9715-5493 Scaffold2514. **c** Farnesyl Diphosphate Synthase: FPPS-1 8816-2271 Scaffold1014368; FPPS-2 6842-3 Scaffold684297. **d** Small Rubber Particle Protein: SRPP-1 43159-46216 Scaffold44842; SRPP-2 13044-16801 Scaffold86876; SRPP-3 1-1958 Scaffold935112

This Meraculous assembly allows characterization of particular genes of interest for guayule improvement, including those involved in rubber biosynthesis. For example, Fig. 2 shows the structures of genes encoding proteins associated with a selected portion on the mevalonate sourced rubber biosynthetic pathway. In general the Meraculous assembly contains the expected isoforms/structures of these genes [30, 31], and encode mRNAs specifically associated with guayule rubber biosynthesis (SRPP-1, Fig. 2) [32].

### Structure of guayule gSaTars

In the course of characterizing the repetitive DNA content in the guayule genomic scaffolds, in particular characterization of non-autonomous Class II TEs, an unexpected pattern emerged. When scaffolds were probed with libraries of small (≤1.7 kbp) terminal inverted repeat elements, clusters of the elements were frequently observed. These clusters had two unexpected properties. First, they were largely associated with microsatellite domains such that individual elements within the cluster are flanked by simple sequence repeats, often (TA)_n_. Second, within the clusters individual elements were modularly assembled, in no case did one microsatellite-flanked element interrupt another. This architecture suggested that the observed modular assembly resulted from the specific targeting of these sequences to microsatellite domains [17, 18, 20, 21]. For this reason, these elements are referred to as *SaTars*, or Satellite Targeted, transposable elements.

**Fig. 3:**
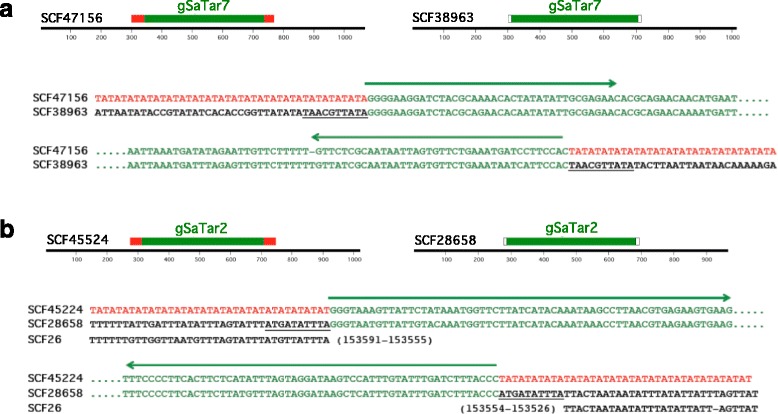
Alignment of *gSaTar* elements flanked by either microsatellite domains or target site duplications in the guayule CLC Genomics Workbench assembly. **a** *gSaTar7* elements. **b** *gSaTar2* elements. Arrows represent terminal inverted repeats. *gSaTar* elements are shown in green, microsatellite domains in red, TSD sequences are underlined. The *gSaTar2* TSD element is aligned to a putative repetitive target site domain from Scaffold26. Sequences shown in alignment: Scaffold47156 311-1379: Scaffold38963 3548-2534: Scaffold45224 275-746: Scaffold28658 4232-4705

The guayule *SaTars*, or *gSaTars*, described here range in size from approximately 400 to 1700 bp, depending on the individual family (Table 2, Additional file 1). As shown in Fig. 3, they have several structural properties common to non-autonomous class II TEs. The *gSaTars* are of appropriate size and are defined by imperfect terminal inverted repeat (TIR) domains (Table 2). Eleven of the fifteen *gSaTar* families contain individual members not localized to microsatellites and flanked by 10 bp target site duplications (TSDs) (Fig. 3, Table 2). In contrast to standard non-autonomous Class II TEs however, a subset of the *gSaTar*s appear preferentially targeted to microsatellite domains (Table 2). Probing (BLAST) the genome with either form (TSD or microsatellite targeted) returns equally elements with both types of insertion events.

**Table 2:** Architecture and distribution of guayule *gSaTar* elements

*gSaTar* element	Size	TIR^a^ length	Flanked microsatellite	Flanked TSD^b^	Linked/Fused *gSaTar*
	bp	bp	%	%	%
*gSaTar1*	990	55	77	6	31
*gSaTar2*	400	40	70	22	19
*gSaTar3*	425	170	77	10	22
*gSaTar4*	400	150	95	4	28
*gSaTar5*	1050	60	80	20	20
*gSaTar6*	500	60	90	4	33
*gSaTar7*	400	50	74	15	21
*gSaTar8*	440	120	87	7	29
*gSaTar9*	520	60	90	0	20
*gSaTar10*	530	55	91	0	27
*gSaTar11*	1030	60	78	0	26
*gSaTar12*	380	180	93	3	47
*gSaTar13*	410	160	94	0	38
*gSaTar14*	1120	250	68	14	37
*gSaTar15*	750	55	62	24	22

^a^Terminal Inverted Repeat

^b^Target Site Duplication

**Fig. 4:**
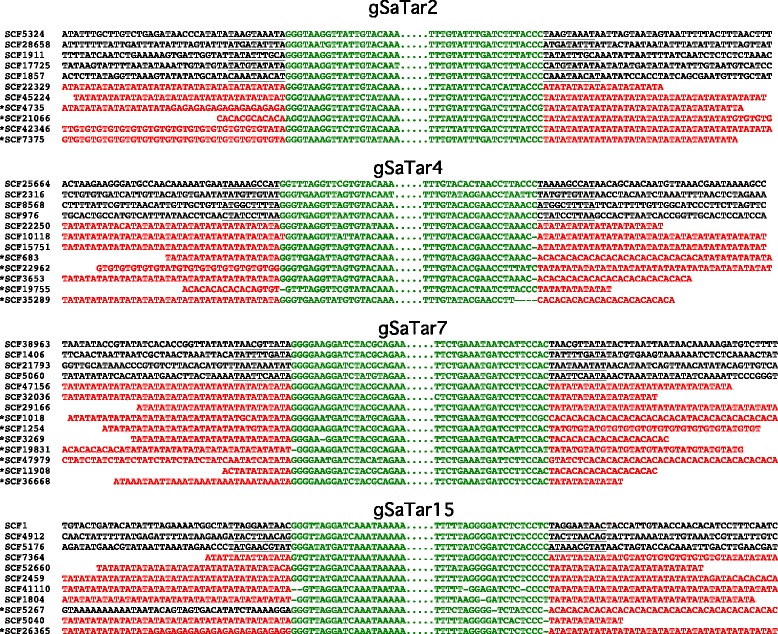
Alignment of *gSaTar* element termini and flanking regions. gSaTar elements are shown in green, microsatellite domains in red, TSD sequences are underlined. The asterisk indicates a flanking structure inconsistent with a 10 bp target site duplication on insertion

While the *gSaTar* elements predominantly localized to (TA)_n_ microsatellite domains, in guayule the type and complexity of the microsatellite domains flanking *gSaTar* elements are highly variable (Fig. 4, for example). Among the *gSaTar* families, at least 78% of the elements are flanked by either microsatellite domains or TSDs (Table 2). The remaining elements, i.e. those with neither architecture, appear to represent loci in which terminal and/or flanking sequences have been altered by local deletions and/or rearrangements.

In Fig. 4, individual members of four *gSaTar* families flanked by either TSDs or microsatellite domains are aligned. In cases where the *gSaTar* elements are flanked by identical microsatellite domains, both structures are consistent with derivation from a straightforward “cut and paste” transpositional mechanism resulting in a 10 bp TSDs. However, as indicated in Fig. 4, the microsatellite sequences immediately flanking the elements are often inconsistent with this mechanism.

The relative frequency of the different *gSaTar* families in the genome are highly variable, and can be estimated by analysis of their distribution on the 16 Mb (1% of genome) artificial assembly (Fig. 1a). This assembly contains 215 gSaTar elements, 32% of which are linked to other *gSaTars* (Additional file 2) indicating the presence of over 20,000 total elements constituting approximately 1% of the guayule genome.

### Genomic distribution of gSaTar elements

**Fig. 5:**
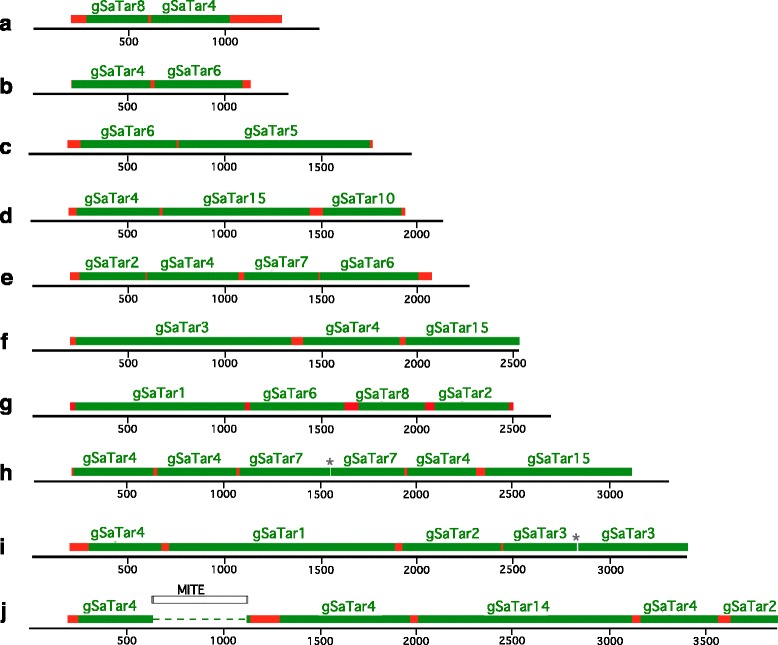
*gSaTar* clusters in the guayule CLC Genomics Workbench assembly. *gSaTar* elements are indicated in green, microsatellite domains in red. Asterisk indicates position of fusion of two *gSaTar* elements resulting from deletion of the linking satellite domain and flanking *gSaTar* sequences. **a** 2387-3884 Scaffold30574. **b** 1885-2024 Scaffold40407. **c** 1482-3426 Scaffold39881. **d** 1214-3350 Scaffold42721. **e** 391-2665 Scaffold54948. **f** 2264-4796 Scaffold 47786. **g** 45799-48502 Scaffold2135. **h** 36014-39336 Scaffold3107. **i** 23170-26571 Scaffold9670. **j** 1138-5004 Scaffold45745

The distribution of *gSaTar* elements along the guayule scaffolds is strikingly different from MITEs and other non-autonomous Class II TEs. MITEs are associated with gene-rich domains in the genome [33, 34]. Specific targeting of MITE insertion events to elements of the same, and different, MITE families results in clusters of these elements [35]. The *gSaTar* elements are also found in clusters on the guayule scaffolds, but with frequency and architecture strikingly different from described in MITEs. In terms of frequency, the *gSaTar* elements are localized to clusters more frequently than observed with the most commonly associated rice MITE elements (Table 2) [35]. And in contrast to the rice-MITE clusters where insertion of MITEs into existing elements is more commonly observed than adjacent insertion [35], the *gSaTar* clusters are always assembled modularly, with intact individual elements linked via microsatellite domains. Examples of *gSaTar* clusters are shown in Fig. 5 (selected cluster domain sequences in Additional file 3). In these clusters, elements from the different families are assembled by adjacent insertions flanked by microsatellites. Within the guayule *gSaTar* clusters internal deletions are observed, some of which remove the intervening satellite sequence in addition to *gSaTar* element ends resulting fusion of elements within the cluster (Fig. 5h and i, for example). However, in cataloging over 300 *gSaTar* clusters, not a single case of one *gSaTar* element interrupting another has been observed, supporting a mobilization mechanism distinct from that involved in the expected non-autonomous Class II TE transposition.

Analysis of one of the *gSaTar* clusters (Scaffold23207) revealed a large, low copy, TA-satellite-linked, repetitive DNA at one end (Additional file 4a). Further characterization indicated that this sequence represents a terminal fragment of a larger defective autonomous *gSaTar-MULE1* (*AgS-MULE1*) *Mu*-like transposon [10], an apparently full length copy of which occurs on scaffold15495 (Additional file 4b). This 5.3 kb MULE element is defined by 400 bp TIRs. A BLAST search of the guayule CLC assembly with the autonomous MULE TIRs returned 5 hits, and in all cases the terminal TIRs were flanked by microsatellite domains. Five additional Autonomous *gSaTar-MULEs* have been identified in the guayule assemblies (Additional file 1). As indicated (Additional file 5), the TIRs defining four of these families are similar to those defining previously described *gSaTar* families (Additional file 1). Both the definition of *gSaTar* elements by long TIRs similar to the *AgS-MULE* elements described above, and the identification of individual members of eleven of the fifteen families flanked by 10 bp TSDs in complex sequence rather than microsatellite domains [36], indicate that the *gSaTar* elements described here represent non-autonomous MULEs that have been specifically targeted to microsatellite domains.

### *SaTar* elements in the rice genome

In order to determine if the *SaTar* mobilization pathway is a general feature of plant genomes, a specific search algorithm for identification of repetitive DNA sequences flanked by microsatellite domains was developed (Methods section). The complete genomic assemblies of rice (*Oryza sativa (japonica) v7_JGI,* [37, 38]) was evaluated employing this algorithm. Five families of rice *SaTars* (*rSaTars*) were returned by the algorithm (Table 3). While the *SaTar* elements are considerably less frequent in rice as compared to the guayule genome (Table 3, Fig. 1b), the *rSaTar* and *gSatar* elements contain a number of common features. Similar to the *gSaTars,* the *rSaTar* families range in size from 380 to 1900 bp. Two resources were employed in the classification of these elements, the RiTE rice transposable element database [36] and a comprehensive listing of rice MULE elements [39]. Four of these families were identified as non-autonomous MULE or Mutator-MITE elements, the most abundant rice TE type (Additional file 6). By comparison to previously mapped rice MULE elements [39], *rSaTar*1, 3 and 5 were further characterized as non-Pack-MULEs. While the diverse MULE element families in the rice genome are largely flanked by 9 bp TSDs [39], only the *rSaTars* in Table 2 were flagged as microsatellite associated by the search algorithm. The final element, *rSaTar2*, was previously identified as small MITE-like mobile elements targeted to (TA)_n_ microsatellites (*Micron*) in rice [17]. However, as shown in Additional file 7, the TIR domains of the *rSaTar2/Micron* elements are highly similar to the TIRs of an autonomous *Mu*-like transposon found on the chromosome one of the *Oryza sativa (japonica)* genome, indicating that this element is also a non-autonomous rice MULE.

**Table 3:** Architecture and distribution of rice *rSaTar* elements

*rSaTar* element	*Oryza sativa v7_JGI*	Size bp	Total elements	Flanked microsatellite	Flanked TSD^a^
*rSatar1 non-Pack-MULE*	Chr2 9609252-9609849	600	36	33	0
*rSaTar2 Micron*	Chr2 16620771-16621153	380	510	492	0
*rSaTar3 non-Pack-MULE*	Chr1 6228684-6229066	380	224	41	155
*rSaTar4*	Chr10 17637383-17638698	1500	176	153	0
*rSaTar5 non-Pack-MULE*	Chr12 21079988-21081881	1900	5	5	0

^a^Target Site Duplication

**Fig. 6:**
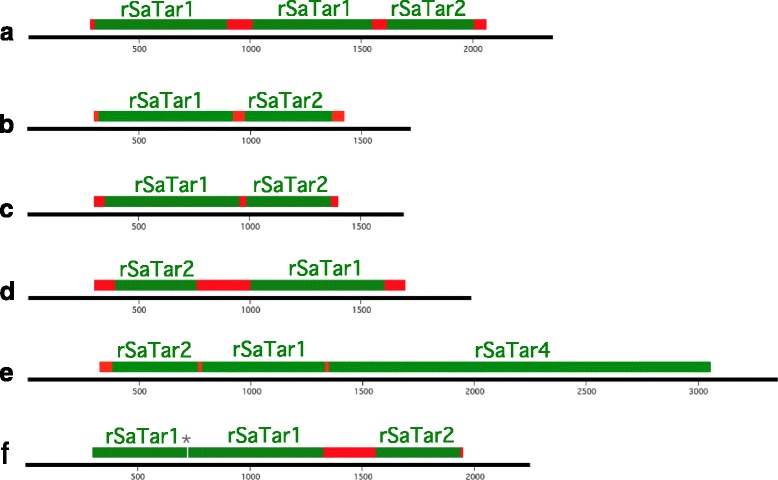
*rSaTar* clusters in the rice (Japonica) genome. *rSaTar* elements, microsatellite domains and deletion indicated as in Fig. 3. **a** Chr2 9.6 = 9608953-9611313, **b** Chr3 15526026-15 **c** Chr3 26142100-26143796, **d** Chr10 1935021-1937019, **e** Chr10 7861025-7862639 linked to *rSaTar*4 interrupted by Ty3, **f** Chr12 4357228-4359474

These *rSaTar* elements share common features with the guayule *gSaTar*s described above (Table 2). First, the *rSaTar*1, 2 and 4 families are defined by TIRs. Second, the elements were frequently associated with microsatellite domains (Table 3). Similar to the four of the *gSaTar* families, the *rSaTar*1, *rSaTar*2, *rSaTar*4 and *rSaTar*5 families had no members flanked by TSDs. In contrast, among the *rSaTar*3 elements, this structure was dominant. In addition, the *rSaTar* elements were frequently clustered (Fig. 6, Additional file 8). For example, of the 36 *rSaTar*1 elements in the rice genome, 6 are linked to *rSaTar*2 elements via microsatellite domains (Fig. 6). Of the *rSaTar*2 elements, 27 are linked to *rSaTar*4 elements (Additional file 8). Finally, in none of the clusters, containing either a single or multiple *rSaTar* types, does one *rSaTar* element interrupt another.

In addition to these non-autonomous *rSaTar* elements, four defective autonomous MULEs which appear to have been mobilized as *rSaTars* (Autonomous rSaTar-MULEs, *ArS-MULEs*) have been identified in the rice genome (Additional file 9). Again similar to the guayule architecture, other *rSaTar* elments are found in the satellite domains flanking these autonomous TEs (Additional files 9 and 10). Of the 12 *ArS-MULE1* elements, 6 are linked to *rSaTar2* elements.

**Fig. 7:**
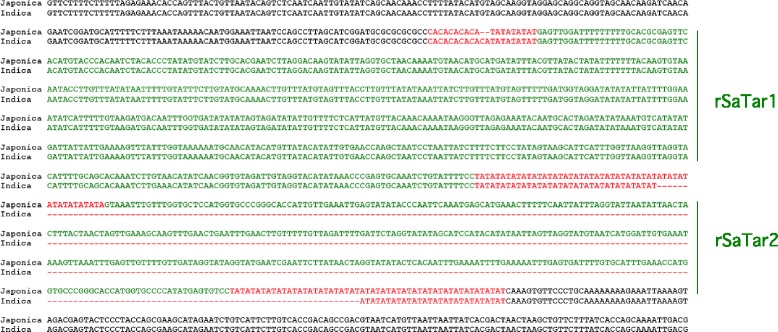
Alignment of rice chromosome 3 sequences indicating an *rSaTar2* insertion in Japonica relative to Indica. Microsatellite domains are indicated in red, *gSaTar* domains are indicated in green. Japonica sequence from *Oryza sativa (japonica) v7_JGI* 15526130-15527603, Indica from *Oryza sativa (indica)* assembly ASM465v1 17276496-17277536

**Fig. 8:**
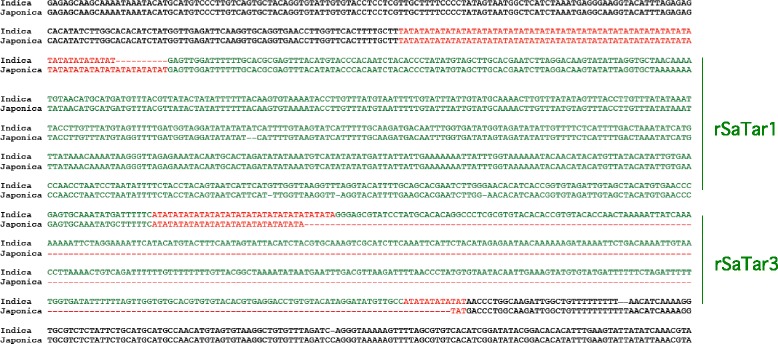
Alignment of rice chromosome 1 sequences indicating an *rSaTar3* insertion in Indica relative to Japonica. Microsatellite domains are indicated in red, *gSaTar* domains are indicated in green. Japonica sequence from *Oryza sativa (japonica) v7_JGI* 20451169-20452242. Indica from *Oryza sativa (indica)* assembly ASM465v1 22805255-22806717

In addition to the *Oryza sativa ssp. japonica* assembly, the availability of a draft genome from the second cultivated subspecies or varietal group, *Oryza sativa ssp. indica* (assembly ASM465v1 [40]) facilitates identification of potential *rSaTar* element insertion sites. These groups diverged relatively recently (approximately 0.44 MYA [41]). Of the 51 *rSaTar*2 sites on *japonica* chromosome 1, 9 are conserved on the *indica* assembly (Additional file 11). In another 20 cases, however, these *japonica* insertion sites are represented by unoccupied TA microsatellite domains of variable length in the *indica* assembly (Fig. 7, Additional file 12). The remainder of the *japonica rSaTar*2 insertion domains are either deleted or unassembled in the *indica* genome. A similar relative *rSaTar3* insertion into an empty satellite flanking an *rSaTar1* element on *japonica* chromosome 3 is shown in Fig. 8. The presence of both conserved and non-conserved *rSaTar*2 loci in *japonica* and *indica* indicates that the *SaTar* targeting system was active before and after the divergence of these two subspecies/varietal groups.

*SaTar*-like elements with structures and distribution profiles were also identified in the sorghum (PhytozomeV9.0:Sbicolor_79 [42], Additional files 13 and 14).

Three families of sorghum *SaTars* (*sSaTars*) were identified employing the algorithm described above (Additional file 13). All three *sSaTar* elements are small (233-373 bp) MITE-like TIR elements. Similar to *rSaTar*1and 2, no *sSaTar*-like elements defined by TSDs were found in the sorghum genome. As indicated in Additional file 13, there are a total of 1261 of the *sSaTar* elements on the sorghum genome, leading to on approximately one element every 600 kb (1261 elements on 730 mb). However, the clustering of these elements is clearly shown in Additional file 14, which shows a cluster of 8 elements on chromosome 1. Degree of clustering of the *sSaTar* elements (Additional file 13) is similar to the guayule *gSaTar* elements (Table 1).

The algorithm employed to identify SaTar elements in rice and sorghum failed to return elements from the citrus genome (Cclementina_182_v1) [43]. However, a manual search of sequences flanking TA satellite domains resulted in identification of three families of small citrus *SaTars* (*cSaTars*) (Additional file 15). The *cSaTar* elements are similar to the guayule, rice and sorghum elements of (definition by TIRs, clustering on chromosomes, modular assembly). In addition, while the majority of these elements are flanked by microsatellite domains, each family contains members flanked by 9 or 10 bp TSDs (Additional file 15). However, the *cSaTar* elements appear relatively ancient as compared to the *gSaTar* and *rSaTar* elements. Examples of *cSaTar* clusters are shown in Additional file 16. Finally, in no cases did the *sSaTar* or the *cSaTar* elements within clusters interrupt each other.

## Discussion

The successful development of guayule as a commercial source of natural rubber is dependent upon improvement of both its agronomic properties and rubber yield. To this end, the sequencing, assembly and annotation of the guayule genome was undertaken to promote the application of current molecular and breeding tools improvement efforts. While fragmented (260 k Scaffolds), the Meraculous assembly reported here is essentially complete allowing, for identification and utilization of specific genes and gene components, as well as contributing to the development of molecular breeding tools (such as genotyping-by-sequencing [5]).

### Guayule *gSaTar* elements are MULE TEs

The *gSaTar* elements were identified in the guayule genome as families of microsatellite-defined, non-autonomous MULE elements with a unique and unexpected chromosomal distribution profile. The MULE designation is based upon structural features of the *gSaTar* elements, characterization of insertion sites and TIR sequence similarity to autonomous guayule MULE transposons.

The *gSaTar* elements are similar to MULE elements in terms of size and structure (defined by TIRs) (Fig. 3, Table 2) [10]. Previously described non-autonomous MULE elements are flanked by 8-11 bp TSDs generated during the “cut and paste” transpositional process [15]. Most of the non-autonomous *gSatar* families described here (Table 2) have individual members flanked by 10 bp TSDs, indicating that these elements are capable of mobilization through a similar pathway. The designation of non-autonomous *gSaTar* elements as MULEs is also supported by TIR sequence similarity to autonomous, microsatellite-targeted, *AgS-MULE* elements that are similarly targeted to microsatellite domains (Additional files 1, 4 and 5). This sequence similarity indicates that the non-autonomous *gSaTars* are mobilized by *AgS-MULE* transposase activities supplied in *trans*.

However, previously described autonomous and non-autonomous MULE elements do not display the sequence-specific (microsatellite domain) targeting exhibited by the *gSaTars* and *AgS-MULEs*. In many cases examination of the structures flanking microsatellite targeted *gSaTar* elements reveals a lack TSDs, these insertion sites are thus inconsistent with insertion via the conventional MULE “cut and paste” mechanism (Fig. 4). The potential utilization of two distinct insertion mechanisms within *gSaTar* families suggests the possibility element subtypes mobilized via independent pathways. However, probing (BLAST) the genome with either form (TSD or microsatellite targeted) returns equally elements with both types of insertion events, indicating that the two distinct insertion profiles do not appear to be associated with specific *gSaTar* subtypes. Rather, the *gSaTar* elements appear to be non-autonomous MULE elements that interact with, or are substrates of, an ancillary system that results in targeted insertion of the mobilized element into microsatellite domains.

The microsatellite targeting of *gSaTar* and *AgS-MULE* elements commonly results in clustering into mixed-family multimers, within which individual elements are present in ordered, continuous arrays of uninterrupted elements. This structure feature was validated through PCR amplification of genomic regions containing *gSaTar* elements in guayule (Additional file 17). The expected nested architecture within these TE multimers, with older component elements interrupted by those more recently inserted [35, 44], was not observed in any of the *gSatar-* or *AgS-MULE*-containing clusters.

Given approximately 20,000 total members of the *gSaTar* families described here, a random distribution would result in approximately 1 element every 80 kb. However, the observed distribution is strikingly non-random. Approximately 30% of the *gSaTar* elements from these families are linked directly to another *gSaTar* element. This value (30%) represents a minimum level of association as our identification of *gSaTar* families is incomplete, and in many cases these elements are located on the scaffold ends where potential association would not be detected (*gSaTar* elements are present at approximately 12% of scaffold ends). Given random insertion, the four linked elements shown in Additional file 3c would be expected to be distributed over approximately 320 kb, not the observed 2.3 kb.

Both the structure and distribution of the *gSaTar* and *AgS-MULE* elements suggest that while they share features with MULEs and Class II TEs in general, and can in cases be mobilized similarly, *gSaTar* transposition involves distinct and/or additional mobilization components. As such, they appear to serve as substrates in a novel mechanism that results in specific targeting to microsatellite domains and modular assembly of diverse elements at specific chromosomal loci.

### *SaTar* elements in other plant genomes

To evaluate whether similar *SaTars* existed in other plant species, rice (*Oryza sativa Japonica*) was selected because of its well defined genome [37], the abundance and diversity of MULE elements [39], and the availability of genomic sequence from *Oryza sativa Indica* [40], a closely related subspecies/varietal group. The rice genome was analyzed employing a specific *SaTar* search algorithm (repetitive DNA sequences flanked by (TA)_n_ microsatellite domains) and five *rSaTar* element families were returned (Table 3). The *rSaTar* elements were similar in size, structure and genomic distribution to the elements from guayule (Fig. 6, Table 3, Additional file 8). These elements could be classified as non-autonomous MULEs by sequence comparison (BLAST) to previously characterized TEs [36, 39], or by identification of an autonomous microsatellite targeted MULE with similar TIRs (Additional file 7). In addition, ancient autonomous, microsatellite-associated rice *ArS-MULE* elements present in *rSaTar* clusters were identified (Additional file 9).

Finally, *SaTar* families in sorghum (*sSaTars*, Additional files 13 and 14) and citrus (*cSaTars*, Additional files 15 and 16) were identified, suggesting that microsatellite-targeting dependent MULE stacking is a general feature of plant genomes. The relative similarity of *SaTar* family members varies considerably in the different species, for example the individual members of the *cSaTar* families have much lower similarity than observed among the *gSaTar*s, indicating that *SaTar* targeting is evolutionarily intermittent.

### *SaTar* targeting

The identification of *SaTar* elements in diverse plant genomic backgrounds offers the potential for an improved characterization of mobilization mechanisms. As discussed above, MULEs, as Class II TEs, are generally mobilized by a transposase-directed “cut and paste” mechanism [45] that results in TSDs, the size of which is diagnostic of the transposons superfamily. As indicated in Fig. 2, individual members of *gSatar* families not targeted to microsatellites are flanked by TSDs expected from *Mu*-superfamily elements (8-11 bp TSDs in low-copy DNA), and are not assembled into arrays of unrelated elements joined end to end. The same is true of the non-targeted *rSaTar3* elements (Table 3). In contrast, the sequences flanking many of the microsatellite-targeted *gSaTar* elements are inconsistent with the “cut and paste” mechanism (Fig. 4) suggesting involvement of a transpositional component distinct from other MULEs.

Given the general instability of microsatellite sequences [16], more recent *SaTar* insertion events would be expected to most accurately retain architectural features generated by the transposition. The TAFTA transposons in maize [20] represent the most recent events of MULE *SaTar*-like insertions described. In these events, empty target sites consisted of short TA microsatellite domains of (6-8 bp) which are expanded following insertion to up to (TA)_50_ on each side of the inserted TAFTA element [20]. While similar apparent microsatellite expansions are easily found on comparison of relative *rSaTar*2 insertions in Japonica and Indica (Figs. 7 and 8, Additional file 12), the innate instability of the microsatellite domains, as well as inability to clearly differentiate *rSaTar*2 relative insertions and deletions, limits the utility of this data. In maize, a TA site-specific transposase was proposed to establish targeting, with expansion of the flanking microsatellite via DNA polymerase stuttering during repair of single stranded gaps [20]. However, in guayule (and other plant genomes, including rice) the *SaTar*-flanking microsatellite domains are often composed of diverse, non-TA, microsatellites (Fig. 4), complicating this targeting model. Rather, the targeting of *SaTars* to a diverse set of different microsatellite repeats suggests involvement of homologous recombination in *SaTar* insertion, not involved with insertion of non-*SaTar* MULE TEs. A separate model for the mechanism of *SaTar* targeting, and thus modular stacking of these TEs, would involve generation of closed circular *SaTar* intermediates [46] with microsatellite domains added as filler sequences between the element termini, followed by homologous recombination into an existing genomic microsatellite target domain. While this model is speculative in nature, the involvement of circular intermediates in MULE transposition has been suggested [12], and circular autonomous elements which include filler sequence have been described [47].

### *SaTars* and gene evolution

While the actual importance of the MULE elements in the evolution of gene and genome architecture remains a topic of investigation [9], both the diversity and distribution of these elements in plant genomes suggests the potential to play an important role as mediators of plant gene evolution, including both coding and regulatory functions [12–15]. The specific targeting of these TEs to microsatellite domains, and the resulting modular assembly of mixed MULE clusters, offer improved evolutionary potential by preventing inactivation of active genes [9, 21] as well as proximally, and modularly, locating sequence divergent elements with the potential to contribute coding and non-coding gene components.

The *SaTar* elements described here do not represent a complete catalog of these elements in any of the selected genomes. However, the described elements are sufficient for demonstrating conservation of *SaTar* structure, chromosomal distribution profiles and cluster architecture among these plant species. It is clear that accurate determinations of both the frequency of *SaTar* clustering and overall contributions to these genomes will require more thorough characterization, and that the values presented here represent minimal estimates of their overall contributions to these genomes.

## Conclusion

We report here the sequencing, assembly and annotation of the guayule genome to provide a foundation for application of modern crop improvement technologies to this plant. In addition, novel non-autonomous MULE *SaTar* elements with unique distribution profiles were identified in this genome, then characterized in other plant species. *Satar* targeting appears based on an alternative MULE recombination mechanism with the potential to impact gene evolution.

## Additional files

Additional file 1: gSaTar terminal inverted repeat sequences. (PDF 55 kb)

Additional file 2: gSaTar elements on the 16 Mb artificial assembly (1% of guayule genome). (PDF 41 kb)

Additional file 3: gSaTar clusters in the guayule CLC Genomics Workbench assembly. (PDF 46 kb)

Additional file 4: Autonomous gSaTar-MULE1 (AgS-MULE1) and cluster with non-autonomous gSaTar elements in the guayule CLC Genomics Workbench assembly. (PDF 47 kb)

Additional file 5: Alignment of non-autonomous gSaTar and autonomous gSaTar-MULE (AgS-MULE) terminal inverted repeat domains. (PDF 69 kb)

Additional file 6: Classification of rice rSaTar elements. (PDF 44 kb)

Additional file 7: The rSaTar2/Micron terminal inverted repeats are similar to those defining the ArS-MULE2 autonomous Mu-like transposon. (PDF 83 kb)

Additional file 8: Sample rSaTar clusters on the rice genome *Oryza sativa* V7_JGI. (PDF 45 kb)

Additional file 9: Autonomous rSaTar-Mules in the rice genome. (PDF 46 kb)

Additional file 10: Autonomous rSaTar-MULE1 (ArS-MULE1) element sequences in the rice (Japonica) genome. (PDF 140 kb)

Additional file 11: Alignment of rSaTar element conserved on Chromosome 1 of rice Japonica and Indica. (PDF 89 kb)

Additional file 12: Sample relative rSaTar insertions in Chromosome 1 of *Oryza sativa* Japonica and Indica. (PDF 45 kb)

Additional file 13: Architecture and distribution of sorghum sSaTar elements. (PDF 56 kb)

Additional file 14: sSatar cluster on sorghum Chromosome 1. (PDF 46 kb)

Additional file 15: Architecture and distribution of citrus cSaTar elements. (PDF 59 kb)

Additional file 16: Sample cSaTar clusters on Citrus. (PDF 35 kb)

Additional file 17: PCR amplification of genomic regions containing gSaTar clusters. (PDF 268 kb)

## Abbreviations

MITEs: Miniature inverted-repeat transposable elements
MULE: *Mu*-like element
SaTar: Satellite Targeted
SSRs: Simple sequence repeats
TE: Transposable element
