# Supplementary material for: Modular assembly of transposable element arrays by microsatellite targeting in the guayule and rice genomes
Source: BMC Genomics. 2018 Apr 19;19:271. doi: 10.1186/s12864-018-4653-6 (PMC5907723; doi:10.1186/s12864-018-4653-6)
Supplement: Supplementary file 1 — gSaTar terminal inverted repeat sequences. (PDF 55 kb) [file 12864_2018_4653_MOESM1_ESM.pdf]

Guayule gSaTar terminal inverted repeat sequences.

gSaTar Element	Size bp	Terminal Inverted Repeat Sequence
gSaTar1a	1150	GGGTAA-GGTTAGTGTACAAAGGGTGTGTTTTGTGAGAACGGTAAGAAG<br>GGGTAGAGGTTTGTGTACAAAAGGTGTTTTTTATGAGAAGTGTAAGAAG
gSaTar1b	800	GGGTAGGGTTATTGTACAAAGGGTGTGTTTTGTAAAAAGGGTAAGAAGTGTT<br>----GTGGTTCGTGTACAAAGGGTGAGATTTGTGAGAAGGGTAAGAAGGATT
gSaTar1c	850	GGATAGGGTTCCTGTATAAAAGGTGTTTTTTTTTAAAAGTGTAAGACGTA<br>----GAGATTCGTGTAC-AAAGGGGTGTTATTGTGAGAAGTGTAAGAAGAA
gSaTar1d	1110	GGGAGAGGCAAGGGATCGGATACAAAATGGAAAAAGTGTGAGAAGTGCAAGAA<br>------GGGGAGAGTTCCTGTACAAAATGTGTTTTTTATGAGAAGGATAAGAA
gSaTar1e	990	GGGTAGGGTTAGTGTACAAAGGGTGTGTTTTGTGAGAATGGTAAGAAGTGTTTTT<br>GGGAAGGGTTCGTGTACAAAGGACCAAAAGTGTGAGAAAGGTAAGAAGTGTTCTT
gSaTar1f	1090	GGGAGGGTTCCTGTACAAAATAGTTTTTTTGTGAGAAATGTA<br>----GGGATCGGGTACAAAATAGAACTTTTGTGAGAAATGTA
gSaTar1fg	870	GGGTTGGGTTAGTGTACAAAGGGTGTTTTTTGTGAGAAGGGTAAGAAG<br>-------GTTCGTGTACAAAGGGTGAGATTTATGAGAAGGGTAAGAAG
gSaTar2a	400	GGGTAAAGATCAAATACAAAAGG-GCTTATCCTATAAACA<br>GGGTAAGGTTCTTGTATAAATGGTTCTTATCCTACAAATA
gSaTar2b	380	GGGTAAGGTTCTTGTACAAATGGAA-TTATCGTACGAA<br>GGGTAAGGTTATTGTACAGTTGGTTCTTATCATACAAA
gSaTar2c	360	GGGTAAGGTTCTTGTACAAATGGAATTATCGTACGAAGCGTACGAA<br>-----GGACAAAAGATCAAATACAA---AGTAAATTATCGTACGAA
gSaTar3a	430	GGGTGGGGTTCGTGTAAAAAGTGTGTTTTTTCTAAGAAGTCTAAGAA<br>GGTAAGGAGTTTGCTAGAAAGTATATTTTTCCTAAAAAGTGTAAGAA
gSaTar3b	390	GGTAA-GGTTGTTGTACAAAGTACATTTTTCGTACAAAGTGTGAGAA<br>GGTAAAGGTTCGTATACAAATTATATTTTTCGTACAAAGTGTGGGAA
gSaTar3c	820	GGGTGGGGTTTCTCTAGAAAGTGGTGTATTTTTAAGAAATT-TAAGAA<br>GGTTAGGAGTTAGCTTGAAAGTGGGGT-TTATCTATAAAGTGTAAGAA
gSaTar4a	400	GGGTGAG-GTTCCTGTACAAAATGGAAAAAGTGTGAGAAATTTTAAAATT<br>GGGAAAGAGATCGGGTACAAAATGAAAAAAGTGTGAGAAATGTGAGAAAT
gSaTar4b	400	GGGTTTAGGTTCGTGTACAAAGGCCATATTTTGTGAGAAGTGTGAGAAGGATTAA<br>GGGTA-AGGTTAGTGTACAAAGGCCATTTTTTGTGAGAAGTGTGAGAAGGATTAA
gSaTar5a	1630	GGGTCAGGATCATGTACAAAAAAAGGTGTTTTGTGAGAAGGGTAAGAAA<br>-----GGGATCTTGTACAAAAA-TTGGGTTTTGTGAGAAGGGTAAGAAA
gSaTar5b	1050	GGGTCAGGATCCTGTACAAATGGTGTTTTTTGTGAAAAGGGTAAGAA<br>-----GGGATCTTGTATAAAAATTAGAAATTGTGAGAAGAGTAAGAA
gSaTar5c	1050	GTAGATGATCCTGTACAGAGTCTAAGATTTGTAAGAAGGGTAAGAAGCCTTGTGGAC<br>---------CCTGTACAAAGGAGGTGTTTTGTGAGAAGGGTAAGAAGGCTTTGTGAC
gSaTar6a	500	GTGGAGAGTTCAAATGAGAAGAAAATGTATTGTAAGAAGAAAAAGAACAAA<br>GGGGA-TGATCAAATAAGAAGAAACT-TAGTGTGAGAATAAAAAGAAGAAA
gSaTar6b	460	GGAGAGTTCAAATGAGAAGAAAAT-ATATCGTAAGAAGAAAAACAAA<br>GGGATAATCAAATGAGAATAAACTCAATGTGAAAGAAGAAAATGACA
gSaTar7	400	GGGGAAGGATCTACGCAAAACACTATATATTGCGAGAACACGCAGAACAA<br>GTGGAAGGATCATTTCAGAACACTAATTATTGCGAGAACAAAAAGAACAA
gSaTar8a	440	GGGGAGGGTTATCTTGAGAACACTAAATATCGCGAGAACCGTGAGAACAAATAAAAA<br>GGGGAGGGTTACTTTGAGAACACTAAATGTCGTGAGAACCGTGAGAACAAATGAAAA
gSaTar8b	400	GGGGAGGGTTATCTTGAGAACGCTAAATATC-GCGAGAACCGTCAGAACAAATGGAAAAA<br>GGGGAGAGTTAATTTGAGAACACATGTATTTTGTGCGAACCGCAAGAACACCGTCTAAAA
gSaTar9	520	GGGTAGGGATCATGCGAGAACCA-ACTTTATTGCGAGAACCGGGAGAACCAATGTGAAC<br>-GATTTGGATCATTTGAGAACCACCATTAATTGTGAGAACCGCGAGAACCAGTGTGAAC
gSaTar10a	530	GGGTAGGGATCCTAAGAGAACCAA-CCCCTATTTGAGAGCCATGAGAACTATTTT<br>-GATTTGGATCATTTGAGAACCAAAGGCCTAATTGAGAACTGAGAAAACCATTCT
gSaTar10b	590	GGGTAGGGATCCTAAGAGAACCAA-CCCCTATTTGAGAACCAAGAGAACCATTCT<br>GGATTTGGATCATTTGAGAACCAATTGGCAATTTGAGAACTGAGAGAACCATTTT
gSaTar11	1030	GGTA-GGGTTCATGAGAGAACCAAGTAAAATGTAAGAACCATAAGAATCAATATGGACC<br>GGATTGGGTTCAAATAAAAACCAAGAATTATATAAGAACCATAAGAACCAATCTAGACC
gSaTar12	380	GGGAAGTTTAAACAGAAAATCCTAAAAATTTT----AGAAAACCCTACTTTCCGAGC<br>GGCTAAGTATAAATAGAAAACCCACTTTTTTTTTGGAGAAAACCCTACTTCCCGAAC
gSaTar13	410	GGGTGAGGATAAAATGAAAACCACCTTGAGTTAAGAAAACCGTT---AAAACCCATTATTTTTTTTTT-ATAAAATTACATG<br>----GAGAAAACCGTGAAAACCA--------TAGCAAAACCCATGTAAAAAAACATTATTATTTTTTTGAAAAAATTACATG
gSaTar14a	1120	GGGAGGTTATTGTACA--AGAGAGCTTGACGTACAAAGTGTAGGAGA<br>GGGGAAGGTTAAAATAGAAGAGAGCTTAACGTACAAAGTGTACAAAA
gSaTar14b	1120	GGGCTGGTTAAAATACAAGATAAATAATCGTGCGAAGAATATGAGACAAT<br>-----GGTTAACATACAAGAAAAATAATCGTGCGAAGGGTAGG-GACAAT
gSaTar15	750	GGGTTAAGATCAAATAAAAAGTTGATTTTGCCTAAGTAGGATAAGAAG<br>GGGGAGAGATCCCCTAAAAATTGGGTTTTGCCTAAGTAGCCTAAGAAG
gSaTar16	840	GGGGAAAGTGAATATGGGGCTGTTACGCATCCAA-GTTGTATGCGAAAC<br>GGGGT----TGTTACGCATCCAAGTTATATGCAA--AACCAATCAAAAC
Autonomous *gSaTar-MULE1*	5330	GGGGAAAGTGAATATGGTGCTGTTGCGCACCTAACCTTACATGCGCAACCATCAAAACTACGTAGTTTTG<br>GGGGAAAGTGAATATGGTACTGTTGCGCACCTAACCTTACATGCGCAACCATCAAAACTACGTAGTTTTG
Autonomous *gSaTar-MULE2*	3850	GGGAAAAGTGAATA--------TGAGGCTGTTAAGTACCCAACCTTACATGTGAAACCATCCAAACTACGTAGTTTTG<br>GGGAAAAGTGAATAAGTGAATATGAGGCTGTTAAGTACCCAACCTTACATGTTGAACCATCAAAACTACGTAGTTTTG
Autonomous gSaTar-MULE3	10800	GGGGAGAGTTCCTGTACAAAATGGATCTTTTGTGAGAAACGTAGGAACCAATCTGCGAGTGACACATGA<br>GGTAGAGGATCGGGTACAAAATGGCTCTTTTGTGAGAAACGTAGGAACCAATCTGCGAGTGACACATGA
Autonomous gSaTar-MULE4	10500	GGGTGGGGTTAGTGTAGAAATGGTGTTTTTTGTGAGAATGGTAGGAATCAAT<br>------GGTTCCGGTGAAAACATGAGTTTTTGTGAGAACAGTGAGACCCAAT
Autonomous gSaTar-MULE5	10800	GGGTAGGGATCCTAAGAGAACCAACCCTAATTTGAGAACCAAGAGAACCATTCT<br>-GGTAGGGATCCTAAGAGAACAAACCTTAATTCGAGAACTAAGAGAACCAATCT
Autonomous gSaTar-MULE6	10100	GGGTAGGGATCATGCGAGAACCA-ACTTTATTGCGAGAACCGCGAGAACCA<br>GGATGTGGATCATTTGAGAACCACACTTTATTGTGAGAACCGCGAGAACCA

Additional file 1.

Terminal inverted repeats defining non-autonomous and autonomous gSaTar elements. Aligned sequences of 5' and 3' (reverse complement shown) defining TIRs.
