# Supplementary material for: Modular assembly of transposable element arrays by microsatellite targeting in the guayule and rice genomes
Source: BMC Genomics. 2018 Apr 19;19:271. doi: 10.1186/s12864-018-4653-6 (PMC5907723; doi:10.1186/s12864-018-4653-6)
Supplement: Supplementary file 2 — gSaTar elements on the 16 Mb artificial assembly (1% of guayule genome). (PDF 41 kb) [file 12864_2018_4653_MOESM2_ESM.pdf]

gSaTar elements on 16 Mb assembly.

gSaTar Element	Elements on 16 Mb Assembly	Linked/Fused *gSaTar*
gSaTar1	24	11
gSaTar2	23	4
gSaTar3	13	5
gSaTar4	39	11
gSaTar5	3	0
gSaTar6	26	11
gSaTar7	9	3
gSaTar8	13	5
gSaTar9	13	3
gSaTar10	21	7
gSaTar11	8	0
gSaTar12	1	1
gSaTar13	5	5
gSaTar14	8	0
gSaTar15	9	3

Additional File 2.
gSaTar elements on the 16 Mb artificial assembly (1% of guayule genome).
Frequency and association of gSaTar elements.
