# Supplementary material for: Modular assembly of transposable element arrays by microsatellite targeting in the guayule and rice genomes
Source: BMC Genomics. 2018 Apr 19;19:271. doi: 10.1186/s12864-018-4653-6 (PMC5907723; doi:10.1186/s12864-018-4653-6)
Supplement: Supplementary file 3 — gSaTar clusters in the guayule CLC Genomics Workbench assembly. (PDF 46 kb) [file 12864_2018_4653_MOESM3_ESM.pdf]

a

```
CGGCTACGGGCTATGTGTTAAAAACAATTTAATAAAATAAAATTCAGAAAATTATATATATATATATATATAT
ATATATATATATATATATATATATATATATATATATATATATATATATATATATATATGGGAGGGTTATTTTGAG
AACGCTAAATATAGCGAGAACCGTGAGAACAAATGGGGAAACCATTCAAAATCAATTTCTTTTAATACAACCT
TCATTATAATTAAAATATAACACTAATAAAAGAATTTTCCTTTTAAATAATTCATCTCCTCTCGCAAAAATAC
TCTAAAATAATATTTTTGTACATGTGTAAATGAAACAGTTTACACATGTGTAAAAACAATTTATTTTTGTATA
CGTTGAAATTTTTTCGCGTAATTTATGTTTTGAGATTGTATTTCAATAATAATGATAAGTGTAAAAAGAAATT
TTAAATTTAAATGGTTTTATATATATATAGGTAAAGGATCATGTACAAAGTCTATGTTTCGTGAGAAGTGTGA
GAAGGATTAAAAATTGACATGTAGGCATCATGTTTTGGATTGAGGCATGATGTTTTTGGTTGTTTTTGCTAGA
AAAATATTAAACACAACAATTTAAAACACAATACAATGTATTATAGCCATGGATTTAAAACACAACTAAAACA
CAAAGCTAAAACACAACAATTTAAAACACATGTTCTTGGCATGGCGTTTTACATTTTAAGCCTAAAATAAATT
GCTTTGTGGTTTTAGTTGTTATGTTTGATACTATTTTAGCCTAAATAATCCGAAACAACATAAAAAAATAAAA
AAAAATGATGTCTACATATCAATTCTTAATCCTTCTCACGCTTCTTACGAAAAATGGCCTTTGTACAGGAACC
TCACCATACACACACACACACACACACACATACATACGTACGTACATACATACATACATACATACATACGTAT
ATGTATGTATATATACATATATACATATATATGTATGTATATGTATGTATATATATATGTACATATATGTATG
TATATATATATATGTATACATATATGTATATGTATGTATATATACATATATACATATATATATATATATATAT
GTATATGTGTATATATATATATATATATATGTATATATATATATATATACATCACAAAATAAAAGTACCAAAA
AAAATCACCAAAAAAAAAATCACAAAAT
```

gSaTar8

gSaTar4

b

```
GCTCTTACAAATTGAATGCGGTTTTCTTATTAATGACATACTAAAACAACATATATATATATATATATATATA
TATATATATATATATAAGGTTAGTGTACAAAGGTCATCTTTTGTGAGAAGGATTAAGAATTGATATATATATA
TATATATGCATCATTTTTTGACTTAGTTATGATGTTTTGGATTGTTTTGGCGATAAAAACACTAAACATAACT
ATTAAAAACACAAATCAATGTATTCTAGACTTAAAACTTAAAACACCATACCAAGAACGTTAAGCAGTGTGTT
TTATGTGGTAAGCAGTGTGTTTTATGTCTTAAGCAGTGTGTTTTATTTGTTTTTTAAGTTTCATGGCTATAAT
ACATTGTATTGTGTTTTCGATTGTTGTGTTTAGTGTTTTTCTAGCAATAACAACCTAAAACATCATGCCTAAA
TTCAAAACATGTTGTCTATATGTCAATTCTTAATCTTTCTTACACCTCTCATAAAATATGGCCTTTGTACACG
AACCTAAACATATATATATAGGGTTAGGATCAAATAAAAAGTTGATTTTGCCTAAGTAGGATAAGAAGCTATC
TTGACCTTTTATTTTTTAAATCAATGACTAAGATGAAAGTGTAGGGAAAAAGGGGGGAGTAAGGGTATTTTT
GGAATAGCTTATTTATTGACTTTCTCTCTCTACATGCAAGACACATGCATATTTTCCCTCCATTTTTTAAACG
GCCATAACTTTTTCACACGACCTTATTTTTTTAAAAAAATTTGCACCATAAAATCGAGCGTCTTTTTATCTAC
GATTTGAGTACCATTTTGCTATATAAGAAACGTCAATTGAAAAAACCCAAAACACGGGTTGTATTTCTATGTT
GTATAACTTTTTTCGTGCTACATTTTTTTGGGCTAAATTTTTTTGTGCTAGATTTTTGTGCTAAAAGTTTTTG
TACTTGATTTTTTTTGTCCCGAACTTTTTATACTAATTTTTTTGTGCCAAATTTTTCGTACTACATTTTTTTG
TATTGGATTTTTTGTTCTAAATTTTTTTGTACTATATTTTTTTGTACTATATTTTTTTGGGCTAGATTTGTTC
TACTATATTTTTGTGTGCTAGATTTTTTGTACTAAATTTTTTGGTACTATATTTTTTTTATGCTTGATTTTTGT
GTTGGATTTTGAGAAAATAAAAGGGGAAAGGTTTATGTCTTTAGACCAAAATACCCTTTAATCTAAATCTAAA
GAGTGTCACATGTCATCTCCACAATACTTTTTAGGCTACTTATGCAAAACCTACTTTATATATATATATATAT
ATATATATATATATATATATATATATATATATATATATATATGTATATATGTATATGTATGATTTGGATCATTTG
AGAATCAATCGGCAATTTGAGAACTGAGAAAACAATTTTGGACCACAAGTTTTCAAAGACAATTTTTTTTTT
TTTTTTGCTAAATCACAGCTTTTCTAGCAAGGGTTTTTTTTTATACATATATGTATATACATGTCTAAAACAA
AAAATAAATACTGTACATATATGTACATATGTTTTGATAATGTACATATATGTGCAAATGTGTAAAAATGATT
ACACATCATGTACAGAAAGAAATATTATGGGTAAGAAAATGTTTTTCTAGTGTACAAACGTGTCTATACGTGT
TTGAATTTGAAAAATAAGTACATACATATGGGTTTGACAATATACGCATATCTATAATTGAAAATTGATGATA
TACACATACGTGTAAATGCAAAAAAAAAAAAAAAAGCAACTGATTGTGATCATTTGAAACTATTTATTTAAAC
TCAGGTGCACAT
```

gSaTar4

gSaTar15

gSaTar10

c

```
CAACCACATATTTCGTAAGATACATAAGAATTTTTACACACATATTAATAATATATATATATATATATATATAT
ATGGTAGGGTTAGTGTACAAAGTGTGTGTTTTGTGAGAAGGGTAAAAAATGTTTTTGGCCATTGGATCTATA
AATGGAGGGTAGAGATTGATCAATGACAAAATTGTAATATGTATTATAATTTTGATTGCCTTATTTGACTAG
ATAGGGATATAATAGTAATTTGGTGTGTTCTAAATTTGAAAACCCACATGCAAGACAAATGTAACTTCCACT
CGTCTTTTTTAAACGTCAATAACTTTTTTATGCGTAATTATTTTAAAAAAAAAATGCACCACAATAACAATC
ATTTTTTTACCTTTAATATGAGTACTATATTGCTATATATATACATATAAAAATTAGATTTAAACTGAATAT
GTTTTGTTATATAAAACACGGTTAACTAGTCTGATTCATTTATAACACACTGAAACTAGTCTAATTCATTTA
AAACACACTGAAACTGGTCTGATTCATTTGAAACACACCTGTTATGTCGGTTAACTATAGTGTGTTTTATAT
GAATCTGATTCGTTTTCATGGTAGGTGTTTTATATCAATCATATGTATTAGATGCTGGTTAGCCATCTCAAA
TGAACAAAACACCCCAGGAAAGAGGATCGAATTTTATCAAGTTGAATGATCAATTTTTTTAAGTTGATGTTA
CGGTTGGTTTGATTATTATATAACGATAAAGATGATATTTTGATTGTAATTTGGATTGATATAAATCCCTAT
AAAATACTTAATCAAATAACTACAATTTGGATCATTGTCAATGATCCCCATAAAATTGTGTTTTATCAAATT
ACCTTAATACCCTCAATCTCTAAATTTAATGATGGTCACATGTCATCACAAGAATCCTTCTTACACTTCTCA
CAAATCTCACCCTTTTTACACGAACCTCTCTCTCTCTATATATATAGTGGAGAGTTCAAATGAGAAGAAAAT
GTATAGTAAGAAGAAAAAAAAACACATTTAAACCAATAGAAATTTTCTATGTTAATAGTTGTATTTAATGTT
AAATAAATTATTAAGTTTTTGTAACTCCTCTAAACTAGTTAAATAAATGAAACTCATAATTCATTTTTAAAA
TATATTATTTTAAACAAACCTAATTTAGAGTGAAGAATAATTTCGATATGTATAGGATATACGTAGGATGAA
TTTTTATATGTGTAGGATAAACGTATGATAATCTCGATATGTGTAGGATAAACATATGACAATTTCAGTATA
TGTAGGATGAAATTTGATATGTGTAGGACAAATTTTGATAATGTGTAAGATAAAGTGTTAATAACCAATTAA
TTAGATAGATAATACTTAATGCAAGAAAAGTTAAAATATTTAATGTTTACCATTATGCCATTTTCTTCTTTT
TATTCTCACACTAAGTTTCTTCTCATTTGATCCCCCCCTATATATATATATATATATATATATATATATATAT
ATATATATATATATATATATATATATATATATATATTATCTTGAGAACGCTAAATATCGCGAGAACCGTGAG
AACAGATGGGAAAACCAATCAAAATCAATTTTTATACAACAATCATTAAAATTAAAATACAACTTAAAAAAA
AAGAAGTTTCCTTACAAATAATTCATCTCTTCTAACAAAAAACACTCTAAATTAATGTTTTTCACATGTGTA
AAAACAATTTATTTTCGTATACGTTGAATTTTTTTTGCGTAATTAGTGTTTTTGAGATTGTATTTAAATAAT
AATAATAAGTTTAAAAAGAAATTTTGAATTTGAATGGTTTTAGATGGTGTTCTCGCGGTTCGCACGAAATAT
ATGTATTCTCAAATTAACGCTCCCCTATATATATATATATATATATATATATATATATATATATATATATATAT
AGGGTAAAGATCAAATACAAATGGGCTTATCCTACTAAACATGAGAAGTGAAGGAAGAATTTTTTTTTTATT
TTTTTTCAAAATTTTTTCCCATCTATTTTAGTATGCTTTCTAGATGAGAAATATACAAAAAAAATTAATTTT
TTTTTTGTGTAATTACGCTAGTAGAGTAATTACAGTAATTACCCTACAAAGTGTAGAGTAATTACTCTACTA
GCGTAATTACGCCCAAAAAAAAACTTTAATTTTTTTTTAGAATTCAAATCTAAAATACATGTTTAACAAGATG
AGAAACAAAAAATTTCGAAAAAAAATAAAATTTTTTTCCCTCTTTCACTTCTCACGTTAAGACTTATTTGTA
AGATAAGAACCATTTGTACAATAACCTTACCCTATATATATATATATATAGACACACACATCATCCATCAAA
CACAAACATTCCATAATATTATTAATATATTTTAAGACACAC
```

gSaTar1

gSaTar6

gSaTar8

gSaTar2

Additional file 3.

gSaTar clusters in the guayule CLC Genomics Workbench assembly. gSaTar elements are indicated in green, microsatellite domains in red.
a. 2534-3728 Scaffold30574 b. 1364-3200 Scaffold42721 c. 45949-48366 Scaffold2135 .
