# Supplementary material for: Modular assembly of transposable element arrays by microsatellite targeting in the guayule and rice genomes
Source: BMC Genomics. 2018 Apr 19;19:271. doi: 10.1186/s12864-018-4653-6 (PMC5907723; doi:10.1186/s12864-018-4653-6)
Supplement: Supplementary file 4 — Autonomous gSaTar-MULE1 (AgS-MULE1) and cluster with non-autonomous gSaTar elements in the guayule CLC Genomics Workbench assembly. (PDF 47 kb) [file 12864_2018_4653_MOESM4_ESM.pdf]

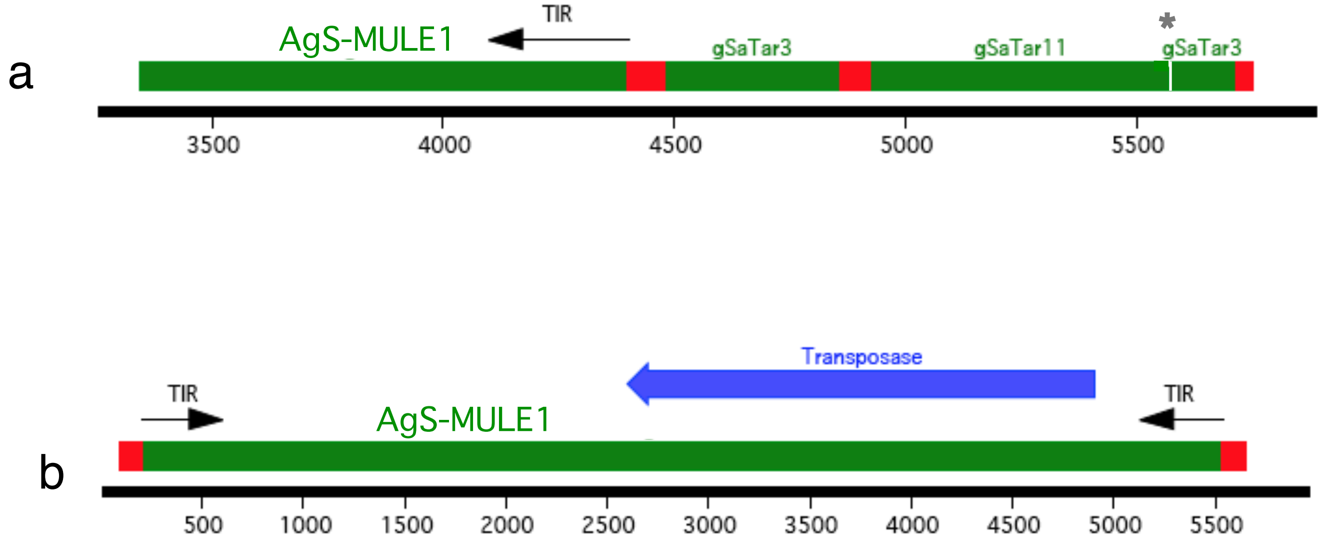

Additional File 4.
Autonomous gSaTar-MULE1 (AgS-MULE1) and cluster with non-autonomous gSaTar elements in the guayule CLC Genomics Workbench assembly. gSaTar and AgS-MULE elements are shown in green, microsatellite domains in red, Terminal Inverted Repeats (TIR) indicated by black arrows, MULE transposase codons indicated by blue arrow, asterisk indicates deletion position gSaTar11 3' - gSaTar3 5' deleted. a. 3211-5751 Scaffold23207. b. 1-5800 Scaffold15495.
