# Supplementary material for: Modular assembly of transposable element arrays by microsatellite targeting in the guayule and rice genomes
Source: BMC Genomics. 2018 Apr 19;19:271. doi: 10.1186/s12864-018-4653-6 (PMC5907723; doi:10.1186/s12864-018-4653-6)
Supplement: Supplementary file 5 — Alignment of non-autonomous gSaTar and autonomous gSaTar-MULE (AgS-MULE) terminal inverted repeat domains. (PDF 69 kb) [file 12864_2018_4653_MOESM5_ESM.pdf]

a

```
AgS-MULE3  TATATATATAGGGGAGAGTTCCTGTACAAAATGGATCTTTTG-GAG-AAACGTA......TACGTTTCTCACAAAAGAGCCATTTTGTACCCGA-TCCTCTACCTACCTACCTACCTATATAT
gSaTar1f              GGGAGGGTTCCTGTACAAAATAGTTTTTTTGTGAG-AAATGTA......TACATTTCTCACAAAAGTTCTATTTTGTACCCGA-TCCC----
gSaTar1c             GGATAGGGTTCCTGTATAAAAGGTGTTTTTTTTTTAAAAGTGTA......TACACTTCTCACAATAACACCCCTTTGTACACGAATCTC----
```

b

```
AgS-MULE4 GTATATATGTAGGGTGGGGTTAGTGTA---GAAATGGTGTTTTTTGTGAGAATGGTAGGAA......TCTCACTGTTCTCACAAAAACTCATGTTTTCACCGGAACCTTTATATATATA
gSaTar5a             GGGTCAGGATCATGTACAAAAAAAGGTGTTTT--GTGAGAAGGGTAAGAA......TCTTACCCTTCTCACAAAACCCAAT-TTTTGTACAAGATCCC
gSaTar5b             GGGTCAGGATCCTGTACAAA---TGGTGTTTTTTGTGAAAAGGGTAAGAA......TCTTACTCTTCTCACAATTTCTAAT-TTTTATACAAGATCCC
```

c

```
AgS-MULE5 TATATATATAGGGTAGGGATCCTAAGAGAACCAACCCTAATTTGAGAACCAAGAGAACCATTCT.....AAGAATGGTTCTCTTGGTTCTCAAATTGCCAATTGGTTCT----------------ATATATAT
gSaTar10b           GGGTAGGGATCCTAAGAGAACCAACCCCTATTTGAGAACCAAGAGAACCATTCT.....AAAAATGGTTCTCTCAGTTCTCAAATTGCCAATTGGTTCTCAAATGATCCAAATCC
gSaTar10a           GGGTAGGGATCCTAAGAGAACCAACCCCTATTTGAGAGCCATGAGAACTATTTT......AGAATGGTTTTCTCAGTTCTCAATTAGGCCTTTGGTTCTCAAATGATCCAAATC-
```

d

```
AgS-MULE6 ATATATATATATTATAGGGTAGGGATCATGCGAGAACCAACTTTATTGCGAGAACCGCGAGAACCAATGT.....TGGTTCTCGCGGTTCTCACAATAAAGTGTGGTTCTCAAATGATCCACATCCTATATATATATA
gSaTar9                   GGGTAGGGATCATGCGAGAACCAACTTTATTGCGAGAACCGGGAGAACCAATGT.....TGGTTCTCGCGGTTCTCACAATTAATGGTGGTTCTCAAATGATCCAAATC-
```

Additional File 5.

Alignment of non-autonomous gSaTar and autonomous gSaTar-MULE (AgS-MULE) terminal inverted repeat domains.

a. Alignment of the terminal inverted repeats defining autonomous AgS-MULE3 (CLC-Scaffold1884 56971-41832). b. Alignment of the terminal inverted repeats defining autonomous AgS-MULE4 (CLC-Scaffold3452 21113-10167). c. Alignment of the terminal inverted repeats defining autonomous AgS-MULE5 (CLC-Scaffold8263 29876-19005). d. Alignment of the terminal inverted repeats defining autonomous AgS-MULE6 (MeraculousScaffold65485 40526-50707). AgS-MULE2 (Scaffold 19101 9877-13725) not shown. gSaTar TIRs from Additional file 1.

.
