# Supplementary material for: Modular assembly of transposable element arrays by microsatellite targeting in the guayule and rice genomes
Source: BMC Genomics. 2018 Apr 19;19:271. doi: 10.1186/s12864-018-4653-6 (PMC5907723; doi:10.1186/s12864-018-4653-6)
Supplement: Supplementary file 6 — Classification of rice rSaTar elements. (PDF 44 kb) [file 12864_2018_4653_MOESM6_ESM.pdf]

Classification of rice *rSaTar* elements.

rSaTar Element	RITE Assignment [32]	MULE Assignment [34]	*Oryza sativa* v7_JGI
rSatar1	Non-Autonomous MULE	non-Pack-MULE Os1274	Chr2 9609252-9609849
rSaTar2	Micropon [16]		Chr2 16620771-16621153
rSaTar3	Non-Autonomous MULE	non-Pack-MULE Os0098	Chr1 6228684-6229066
rSaTar4a	MITE-MuDr		Chr10 17637383-17638698
rSaTar4b	MITE Unknown Transposon		Chr2 20303841-20305665
rSaTar4c	MITE-MuDr		Chr7 14145183-14147066
rSaTar5	Non-Autonomous MULE	non-Pack-MULE Os1961	Chr12 21079988-21081881

Additional File 6.
Classification of rice rSaTar elements by sequence comparison to the available database.
Indicated rSaTar sequences used to probe (BLAST) databases.
