# Supplementary material for: Modular assembly of transposable element arrays by microsatellite targeting in the guayule and rice genomes
Source: BMC Genomics. 2018 Apr 19;19:271. doi: 10.1186/s12864-018-4653-6 (PMC5907723; doi:10.1186/s12864-018-4653-6)
Supplement: Supplementary file 7 — The rSaTar2/Micron terminal inverted repeats are similar to those defining the ArS-MULE2 autonomous Mu-like transposon. (PDF 83 kb) [file 12864_2018_4653_MOESM7_ESM.pdf]

a

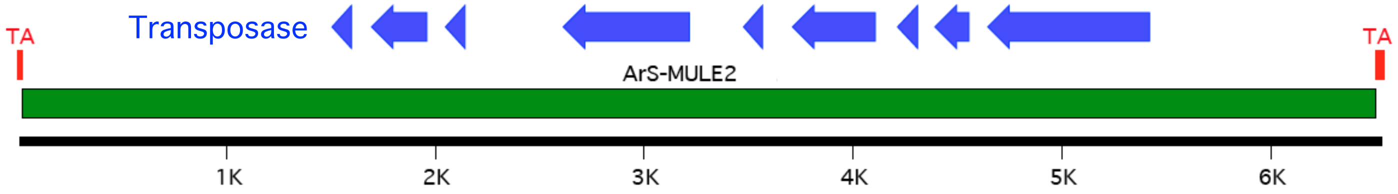

b

```
ArS-MULE2a    TATATATATATATATATATAGGACGCCCAGATAGGACTCCATGGTGCCCGGGCACCA..6367 bp..ATGGAGCCCGGGCACCATGGTGCCCCTAACAAATTTACTATATATATATATATATATA
rSaTar2*      TATATATATATATATATATAGGACACTCATATGGGGCACCATGGTGCCCGGGCACCA..318 bp...ATGGTGCCCGGGCACCATGGAGCACCAAACAAATTTACTATATATATATATATATATA
rSaTar2**     TATATATATATATATATATAGGACACTCATATGGGGCACCATGGTGCCCGGGCACCA..318 bp...ATGGTGCCCGGGCACCATGGAGCACCAAACAAATTTACTATATATATATATATATATA
rSaTar2***    TATATATATATATATATATAGGACACTCATATGGGGCACCATGGTGCCCGGGCACCA..318 bp...ATGGTGCCCGGGCACCATGGAGCACCAAACAAATTTACTATATATATATATATATATA
```

c

```
MPRLPKCASVRRNVKRRSIHDDDDFVTPPTRKKAVRVKFVAPVSRCVPSSITPIIQRFSAEKKELVAEMGFEGLLHMPLITNHRDFS
YWLLSRVSPTRSAIELESGEVLPLSPEDVKKVLGIPCVGEELQAPNVEFVSHVKRLIAERMKVASFKDVNKEVLEDVLDKHHRGQMS
KAEKESFKTAFLMLVMMKFLAPQSNMDNKCPRYFSALLDPNEIAKFNWSGYVLNEILDAAATVQKKHADGTKCGYITGCSIYLQVHY
LDNVDFGGDLNLEHNIFPRIALYDRDTIKERYQLDISERNYYQATQFGKLKLRPKNEVCYKRTCERTSVTDSGGNENVQIESQKAQE
LKSFWISCIGNLDKATESLVKVTEASKNMPSVTIPISDEVDVSKGYTCTTVNDDAENGIEVNQTPSLGNDVEDVSAAAANCTPMVHT
HNLKEESIDLNRTVPHRTYTGLDFDPPSFDLNIDYSPSNNTVQVDKDEEDYKCVCKLFEHMGILCCHVIKVMTHIGCRSIPDKYILR
RWSISAREGSGSNLESFRNDKDAASSRTHRHTLLYHACIDLSKQGDISVDAFHLAMKHITDALDEIKLVAGTNESPMGYTDPPNDEP
AGPDQGGEQNEDPAAHQAVQSLPLPPDRRAKRGRPRSTRLKSPVDGGVRKKRICSNCHLKGHYRTGCPTNPENTSKNKVIKVCTKCK
LAGHYASTCGVDTIATYKEQNNTTLLTSGALSGSRQQHISVPVAMDKMNTGAWSDKIDQSLQKYDHPRCRWHILFGTKSELHNLYHA
RTASDFEDTWTALVNEYGLQEENAYLQKAQMLIDDETKPNDSQEIDTDRKDVKREVEAPKLIISRHSDDKFSS*
```

Additional file 7.

The rSaTar2/Micron terminal inverted repeats are similar to those defining the ArS-MULE2 autonomous Mu-like transposon. a. Map of ArS-MULE2 autonomous Mu-like transposon (Oryza sativa (japonica) v7_JGI chromosome 1 2823514-2830054). Transposase coding domains indicated by blue arrows. b. Alignment of the terminal inverted repeats defining ArS-MULE2 and rSaTar2/Micron elements. c. ArS-MULE2 transposase translated sequence (GenBank- BAF03959). SWIM Ziinc Finger (Znf_PMZ mutator type) indicated in red..
