# Supplementary material for: Modular assembly of transposable element arrays by microsatellite targeting in the guayule and rice genomes
Source: BMC Genomics. 2018 Apr 19;19:271. doi: 10.1186/s12864-018-4653-6 (PMC5907723; doi:10.1186/s12864-018-4653-6)
Supplement: Supplementary file 8 — Sample rSaTar clusters on the rice genome Oryza sativa V7_JGI. (PDF 45 kb) [file 12864_2018_4653_MOESM8_ESM.pdf]

Sample *rSaTar* clusters on the rice genome *Oryza sativa* v7_JGI

Chromosome	Location	*rSaTar* Cluster
1	21833407-21835668	*rSaTar4* link *rSaTar2*
2	10098045:10099953	*rSaTar4* link *rSaTar2*
2	16053460:16056756	*rSaTar4* fuse *rSaTar4* link *rSaTar2*
2	34162756-34163649	*rSaTar1* fuse *rSaTar3*
2	34856374-34858294	*rSaTar2* link *rSaTar4*
3	20438629-20440774	*rSaTar2* link *rSaTar4*
3	26142400-26143496	*rSaTar1* link rSaTar2
4	15696975:15698157	*rSaTar1* link *rSaTar3*
4	19516826:19519122	*rSaTar2* link *rSaTar4*
5	12567616-12572059	*rSaTar2* link *rSaTar4* link *rSaTar2* link *rSaTar4*
6	15985917-15988435	*rSaTar2* link *rSaTar4*
6	20319255-20322209	*rSaTar2* link *rSaTar4*
7	9506097-9506985	rSaTar2 link rSaTar3
8	9960768-9964706	*rSaTar4* link *rSaTar5* link *rSatar2*
8	22329781-22332245	*rSaTar2* link *rSaTar4*
9	3172916-3178336	*rSaTar2* link *rSaTar4* fuse *rSaTar4* *fuse* rSaTar4
9	8954399-8957858	*rSaTar4* link *rSaTar5*
10	2068031-2069176	*rSaTar3* link *rSaTar2* link *rSaTar2*
10	3604760-3611878	*rSaTar4* link *rSaTar4* link *rSaTar4* link *rSaTar2*
11	9641607-9643456	*rSaTar2* link *rSaTar4*
11	11762359:11764105	*rSaTar2* link *rSaTar4*
11	11821583-12823391	*rSaTar3* link *rSaTar4*
12	6465083-6466754	*rSaTar2* link *rSaTar4*
12	12446902-12449128	*rSaTar2* link *rSaTar4* link *rSaTar2*

Additional File 8.
Sample rSaTar clusters on the rice genome. Chromosme, location (Oryza sativa v7_JGI) and composition of rice rSaTar clusters are indicated.
