# Supplementary material for: Modular assembly of transposable element arrays by microsatellite targeting in the guayule and rice genomes
Source: BMC Genomics. 2018 Apr 19;19:271. doi: 10.1186/s12864-018-4653-6 (PMC5907723; doi:10.1186/s12864-018-4653-6)
Supplement: Supplementary file 9 — Autonomous rSaTar-Mules in the rice genome. (PDF 46 kb) [file 12864_2018_4653_MOESM9_ESM.pdf]

Autonomous rSaTar-Mules in the rice genome.

Autonomous *rSaTar*	*Oryza sativa* v7_JGI v7	MULE Assignment [34]	Link *rSaTar*
Autonomous *rSaTar-MULE1*	Chr1 20826405-20834624	Os1819	rSaTar2
Autonomous *rSaTar-MULE1*	Chr2 18811452-18821870	Os0284	rSaTar2
Autonomous *rSaTar-MULE1*	Chr3 6597328-6606701	Os1819	rSaTar2
Autonomous *rSaTar-MULE1*	Ch4 2092332-2101810	Os1819	
Autonomous *rSaTar-MULE1*	Chr5 23167711-23177078	Os1819	
Autonomous *rSaTar-MULE1*	Chr6 254885558-25497928	Os1819	rSaTar2, rSaTar3
Autonomous *rSaTar-MULE1*	Chr6 25573986-25582391	Os1819	
Autonomous *rSaTar-MULE1*	Chr7 2848052-2851182	Os1819	rSaTar2
Autonomous *rSaTar-MULE1*	Chr8 1217966-1218881	Os1819	rSaTar2
Autonomous *rSaTar-MULE1*	Chr9 9376655-9377090	Os1819	
Autonomous *rSaTar-MULE1*	Chr9 10393884-10394640	Os1819	
Autonomous *rSaTar-MULE1*	Chr9 16514025-16514464		
Autonomous *rSaTar-MULE2*	Chr1 2823539-2830002		
Autonomous *rSaTar-MULE2*	Chr1 37878844-37880691		rSaTar2
Autonomous *rSaTar-MULE2*	Chr5 12564785-12567593		rSaTar2, rSaTar2
Autonomous *rSaTar-MULE2*	Chr5 16634190-16639278		
Autonomous *rSaTar-MULE2*	Chr6 30572133-30575617		
Autonomous *rSaTar-MULE2*	Chr6 30595763-30599483		
Autonomous *rSaTar-MULE2*	Chr6 30612156-30615549		
Autonomous *rSaTar-MULE3*	Chr1 10767306-10777283	Os2089	rSaTar2
Autonomous *rSaTar-MULE3*	Chr1 36512324-36522301	Os2089	
Autonomous *rSaTar-MULE3*	Ch2 24806996-24815508	Os2089	
Autonomous *rSaTar-MULE3*	Chr2 29038971-29052832		
Autonomous *rSaTar-MULE3*	Chr4 16919269-16929871	Os2089	
Autonomous *rSaTar-MULE3*	Chr4 32643159-32653114	Os2089	
Autonomous *rSaTar-MULE3*	Chr5 21975100-21985078	Os2089	
Autonomous *rSaTar-MULE3*	Chr7 19070055-19079162		
Autonomous *rSaTar-MULE3*	Chr11 9715546-9724257		
Autonomous *rSaTar-MULE3*	Chr11 11685590-11688752		
Autonomous *rSaTar-MULE4*	Chr6 14975979-15039960		
Autonomous *rSaTar-MULE4*	Chr6 15991109-16006998		rSaTar4
Autonomous *rSaTar-MULE4*	Chr7 28038008-28043311		rSaTar2
Autonomous *rSaTar-MULE4*	Chr12 10975025-10983698		

Additional File 9.

Autonomous rice rSaTar element locations and association with non-autonomous rSaTar elements. Locations of defective autonomous rSaTar elements (Oryza sativa v7_JGI) and linked/fused non-autonmous rSaTar elements are indicated.
