# Supplementary material for: Modular assembly of transposable element arrays by microsatellite targeting in the guayule and rice genomes
Source: BMC Genomics. 2018 Apr 19;19:271. doi: 10.1186/s12864-018-4653-6 (PMC5907723; doi:10.1186/s12864-018-4653-6)
Supplement: Supplementary file 10 — Autonomous rSaTar-MULE1 (ArS-MULE1) element sequences in the rice (Japonica) genome. (PDF 140 kb) [file 12864_2018_4653_MOESM10_ESM.pdf]

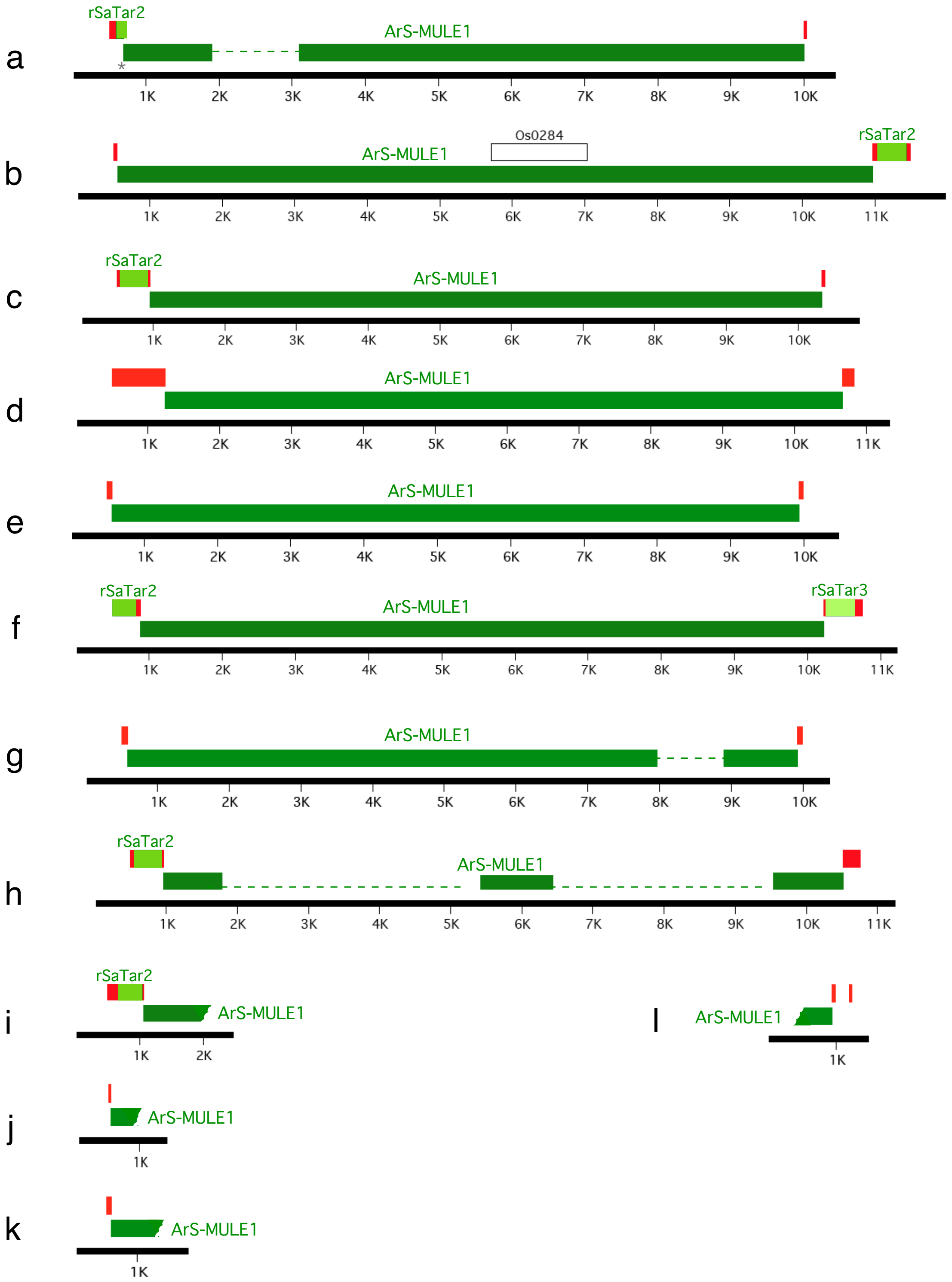

Additional File 10.

Autonomous rSaTar-MULE1 (ArS-MULE1) element sequences in the rice (Japonica) genome. rSaTar, ArS-MULE1, and microsatellite domains indicated as in Additional file 5. a. Chr1, 20825819 - 20835152. b. Chr2 18810899:18822891. c. Chr3 6596779:6607659 RC. d. Chr4 2091088:2102423. e. Chr5 23167147:23177638. f. Chr6 25487562:25498806. g. Chr6 25573405-25582861. h. Chr7 2847086- 2851932. i. Chr8 1216896:1219380. j. Chr9 9376118:9377588. k.Chr9 10393296:10395140. l. Chr9 16513525:16515014.
