# Supplementary material for: Modular assembly of transposable element arrays by microsatellite targeting in the guayule and rice genomes
Source: BMC Genomics. 2018 Apr 19;19:271. doi: 10.1186/s12864-018-4653-6 (PMC5907723; doi:10.1186/s12864-018-4653-6)
Supplement: Supplementary file 12 — Sample relative rSaTar insertions in Chromosome 1 of Oryza sativa Japonica and Indica. (PDF 45 kb) [file 12864_2018_4653_MOESM12_ESM.pdf]

Sample relative *rSaTar2* insertions in Chromosome 1 of *Oryza sativa* Japonica and Indica.

| *rSaTar2* Insertion | *Oryza sativa Japonica* v7_JGI v7 Chr1 | *Oryza sativa Indica* ASM465v1 Chr1 |
|---|---|---|
| Japonica Chr1 | 17754239:17754631 | 19616476:19616515 |
| Japonica Chr1 | 39837306:39837485 | 43540046:43540085 |
| Japonica Chr1 | 40525340:40525701 | 44316689:44316740 |
| Japonica Chr1 | 41775251:41775643 | 45800225:45800266 |
| Japonica Chr1 | 43048589:43048981 | 47071309:47071343 |
| Indica Chr1 | 1655372:1655461 | 2011665:2012059 |
| Indica Chr1 | 6310390:6310414 | 6756065:6756439 |
| Indica Chr1 | 26867078:26867161 | 30057889:30058280 |

**Additional File 12.**
**Sample relative rSaTar2 insertions in chromosome 1 of Oryza sativa Japonica and Indica.**
Locations of the rSaTar2 element and associated empty satellite domain are indicated.
