# Supplementary material for: Modular assembly of transposable element arrays by microsatellite targeting in the guayule and rice genomes
Source: BMC Genomics. 2018 Apr 19;19:271. doi: 10.1186/s12864-018-4653-6 (PMC5907723; doi:10.1186/s12864-018-4653-6)
Supplement: Supplementary file 13 — Architecture and distribution of sorghum sSaTar elements. (PDF 56 kb) [file 12864_2018_4653_MOESM13_ESM.pdf]

Architecture and distribution of sorghum *sSaTar* elements.

sSaTar Element	*PhytozomeV9.0: Sbicolor_79*	Size bp	Total Elements	Flanked Microsatellie	Flanked TSD [a]	Linked/Fused s*SaTar*
sSaTar1	*Chr1 18042758:18042990*	233	509	442	0	227
sSaTar2	*Chr1 57462409: 57462037*	373	522	422	0	183
sSaTar3	Chr1 43739246:43739510	265	230	206	0	91

[a] Target Site Duplication

Additional file 13.
Definition and distribution of sorghum sSaTar elements. Sequences defining individual sSaTar familes are indicated in PhytozomeV9.0:Sbicolor_79 [37]. Total Elements indicates elements in entire assembly.
