# Supplementary material for: Modular assembly of transposable element arrays by microsatellite targeting in the guayule and rice genomes
Source: BMC Genomics. 2018 Apr 19;19:271. doi: 10.1186/s12864-018-4653-6 (PMC5907723; doi:10.1186/s12864-018-4653-6)
Supplement: Supplementary file 14 — sSatar cluster on sorghum Chromosome 1. (PDF 46 kb) [file 12864_2018_4653_MOESM14_ESM.pdf]

```
ACTTATTTATCCATTATGATGCTTGATCCATACTTATCATTATATATATATGCTATTCTACA
CCCTCCGCAAGTCAAAACTAAGTAGAAAAATATTGAGCAATACTGGAGAACCTTCACTATCA   sSaTar1
TTCCGTCCAACACCTAGGACCATGAAATACTGAACAATATTGAAATATGAATACTGAACGAT
ACTGAATTTTCCTCAGTATAACATTTCGATGTAGAATAGTATTCTACATCTAGGGTGTAGAA
TAGCACTTATATATATATATATATATATAGCCAGCTACAAAATAAGTAGTTATTCTGTAGCC
TCCTCTATTTACGATAATTTTATATGCTAATTAACGATAATGGCAATACATATTTACAATAG
TTGGGTTACTATAATACATGGAGATATTTACCATAACGTTATAGTAAATCACTTAGTATGGA   sSaTar3
GTTACTATAATCTCGTAAATTAATATAGTAATTATCGTAACTCACAGTGGCTACAGAATAAG
TTATTTTGTAGCCAGCTACAGAGTGATAGTTATATATATATACGGCAACGTTATTCTACACC
TTAGTGTAGAATATTATTCTACACCGCAAGTCAAATTTGAGTAGAAAAAATACTAAGCAATA
CTGGAGAGTGTTCAGTATCATCTTGTCCAACACTCAAGACCATAAAATACTGAACAATACTG   sSaTar1
AAATATGAATACTGAACGATACTGAATTTTGCTCAGTATAACAGTTTGGTGTAGAATAGTAT
TCTACACCTAGGGTGTAGAATAGCAGTTATATATATATAGGATAACTCCTTTTTACTCCCGG
TAGTAGTTACTCCCATGTATATATCTCTAGATTTATGACGTATATGACCCGATGGAGTATAT
GTAGACGAAGTATATGATCATACTAAATTATCTAGGTAGTATGTATGTTAAGTATGCATGCA
TACATAGAGTATGTGATTATACTTATTATATATACTACAATAGTGACATTTTAATAGTATAT   sSaTar2
TAAAAAATATGAATTTCTTTGAAAATTTTTCATGCGGTAAGATCTAGAGTATCTTATATATA
AACATACTCAAAAAAGTATAAATACATACGTAATATGGTTTATTAAAGATGAGAGTAATTAC
TCCTAAAAGTACGGAAAATGCATCCTATATATATAGGGAAATTCCTTTGTACACGTACGTGT
AGTTACTCTCATCTTCATAAACTACATTGTGTATGTATTCATACTTGTTTATCAGTTTGAAT
ATGTTTATATACTCATATGAAGATACTCAGGATCTTACCACGCGAAAATTTTTCAAAAAAAA
TTATATTTTAAATATATTACTAAAATGCCACTACTATATTATATACAATAAGTATAACCATA   sSaTar2
TACTCTATGTATGTATGCATACTTAACATACATATCACTCTAGATGGTCTAGTATGATCATA
TACTTTGTCTATATATATTTCGTCCGGTCATATACACCTTAAATCTAGAGATACATAGATGA
GAGTAAGTACACGCGCGTGTAAAAGAAACGCGTATATATATATATATATATATATATATATA
TATATATATATATATATATATATATATATATATATATATAACTATTATTATGTAGCTAGCTA
CAAAATAACTTATTCTATAGCCACTTTGAGTTATGATAATTACTACATTAGTTTACGAGATT
ATAGTAACTCTATACTAAGTGGTTGACTATAATATTATGGTAAATATCTCTGTTTAACCCAA   sSaTar3
CTGTCTTAAATATATATAGATATTATCGTAAATTAGTATATAAAATTATTGTAAATGGACAT
GACTATAGAATAACTTATTCTGTAGCTGACTACTGAATATACTCTCCCTATATATATAAGTG
CTATTCTACACCAAACTGTTATACTAAACAGAATTCAGTATCATTCGGTACTCGCGTTTTAG
TGTTGTTCAGTATTTCGTGGTCCTCAGAATAATATTGGATGATACTGAACAATATTCAGTAC   sSaTar1
TACTTAGTAGGTTTCCAGCTCAGTTTTGACTTACGATATAGAATAATATTCTGTAGCTAAGG
TGTAGTATATGGTAGTGCTATTCTACACCCTAGGTGTAAAATTCTACACCAGAAAAAATACT
GAGCAGTACTGAATCGCGTTCAAAATCATCTAATACTACACTCAGGACCATGAAAATACTGA   sSaTar1
ACAATACTGAAACACGAATACTAAACGATACTGAATTCTGCTCAATATAACAATTCGATGTA
AAATTCTACACCTAGGATGTATATATATATATACTAGCATATGCCCGTGCGTTGCTACGGAA
ATAAAATTTCAATGCAACAATGATATAAAAACTATGTGCCATCAAAATATATAGACAAACAT
```

**Additional file 14.**
**sSaTar cluster on sorghum Chrmosome 1.** sSaTar elements are indicated in green, microsatellite domains in red. PhytozomeV9.0:Sbicolor_79 [37] Chromosome 1 22152004-22154359.
