# Supplementary material for: Modular assembly of transposable element arrays by microsatellite targeting in the guayule and rice genomes
Source: BMC Genomics. 2018 Apr 19;19:271. doi: 10.1186/s12864-018-4653-6 (PMC5907723; doi:10.1186/s12864-018-4653-6)
Supplement: Supplementary file 15 — Architecture and distribution of citrus cSaTar elements. (PDF 59 kb) [file 12864_2018_4653_MOESM15_ESM.pdf]

Architecture and distribution of citrus *cSaTar* elements.

cSaTar Element	*C. clementina v1.0*	Size bp	Total Elements	Flanked Microsatellie	Flanked TSD[a]	Linked/Fused c*SaTar*
cSaTar1	Chr5 31167675:31167909	236	33	28	3	10
cSaTar2	Chr2 3400505:3400664	160	166	131	4	35
cSaTar3	Chr3 12239500:12239322	180	91	66	3	9

[a] Target Site Duplication

Additional file 15.

Definition and distribution of citrus cSaTar elements. Sequences defining individual sSaTar familes are indicated in Cclementina_182_v1. Total Elements indicates elements in entire assembly.
