# Supplementary material for: Modular assembly of transposable element arrays by microsatellite targeting in the guayule and rice genomes
Source: BMC Genomics. 2018 Apr 19;19:271. doi: 10.1186/s12864-018-4653-6 (PMC5907723; doi:10.1186/s12864-018-4653-6)
Supplement: Supplementary file 16 — Sample cSaTar clusters on Citrus. (PDF 35 kb) [file 12864_2018_4653_MOESM16_ESM.pdf]

a

```
TTAGCAGACGTGATGAGTTTATATTACTAATTTTTACCAGTTATTCAG
CCATATATATATATATATAGTTTGCTGGTCAGTGATTTTAAAGTGCGGGA
AAACTTTAAAACCACATGACTTTAAAAGCTTAAAACTGTAAGTCTTTA    cSaTar2
AAGCCGTGCGGTTTTAAAGCTTTACCACACTTTCCCGCACTTTAAAAA
TCTGGACTGTAACATTTATATATATATATATAGTAGTTTTTCAATCAAGG
ATTAGCTAAATGACAAAATAAACACAGACTCCAATGCACTATATTTTG
TCAGGTAATGTATTAAAATCTAATTTTTTTTTTTGTCTTTTAACTGATC    cSaTar3
CCTGACTTTATTAAAGTCAGGGATCCCTGATTGAAAAACTATATATAT
TAAAAATGTTCAAATCTGGAATTTTAAAGTGTGAGAATGTCCAGGAAA
ATACATAAACTGTGCAGTTTAAGCGCTTTAAGTTGTAAACCTTAAAAC    cSaTar2
CCCGCAGTTTTAAAGTTTTTCTAGATATTTCCCGCACTTTATGATTCC
GGACCAGAAAACTACTGAATATATATATATATATGATTAAATTATTCTCT
TACGTTTAGGCGCAAAATAGTTGTTTTGTCAATA
```

b

```
ATTAACGTTCTTACTTTAATAAAGTACAAAAAACCTCATCAGATAACAA
CTATATATATATATATATATATATACATATATATAAATATAATTCTATAAT
TCAAAATTTTAAAATGCAAAAATGTTCAGAAAAACGCATAAATCTTGTG    cSaTar2
ATTTAAACTTTTTAAGTTTTAAACCTTTAAAACTCCGCGGTTTTAAAGT
TTTCTAAATGTTTCCCATTCTTTATGATTTCGGACTACAAAATTATATA
TATATATAGTGCTGTTCCAATCCGGGATCCCGGATTGGAACAATGTTCG
GGAGGGAGGCTGGTGGCCTTTGGATTCAAATCCAAAGGCCACCTGTGGC    cSaTar1
TGCACATGGTGGAGCCCACCATGTGCAGCCAAACCAAAAAAATTAAAAG
AAGAAATTTGGGGTCGTTGGACTTGAATCCAACGACCACAAATTTCTTC
CCGGATGGTGTGTATGTATGTATGTATGTATGTAGTGTGGCCCTCCTCA
CTTTATTAAAATGAGGAAAAACACTAAAATATAAAAAAAAAAAACA
```

Additional file 16.
Sample cSaTar clusters on Citrus. cSaTar elements are indicated in green, microsatellite domains in red. a. Cclementina_182_v1 chromosome 2 12457999-12455998. b. citrus Cclementina_182_v1 chromosome 3 35425059-35425593.
