# Supplementary material for: Modular assembly of transposable element arrays by microsatellite targeting in the guayule and rice genomes
Source: BMC Genomics. 2018 Apr 19;19:271. doi: 10.1186/s12864-018-4653-6 (PMC5907723; doi:10.1186/s12864-018-4653-6)
Supplement: Supplementary file 17 — PCR amplification of genomic regions containing gSaTar clusters. (PDF 268 kb) [file 12864_2018_4653_MOESM17_ESM.pdf]

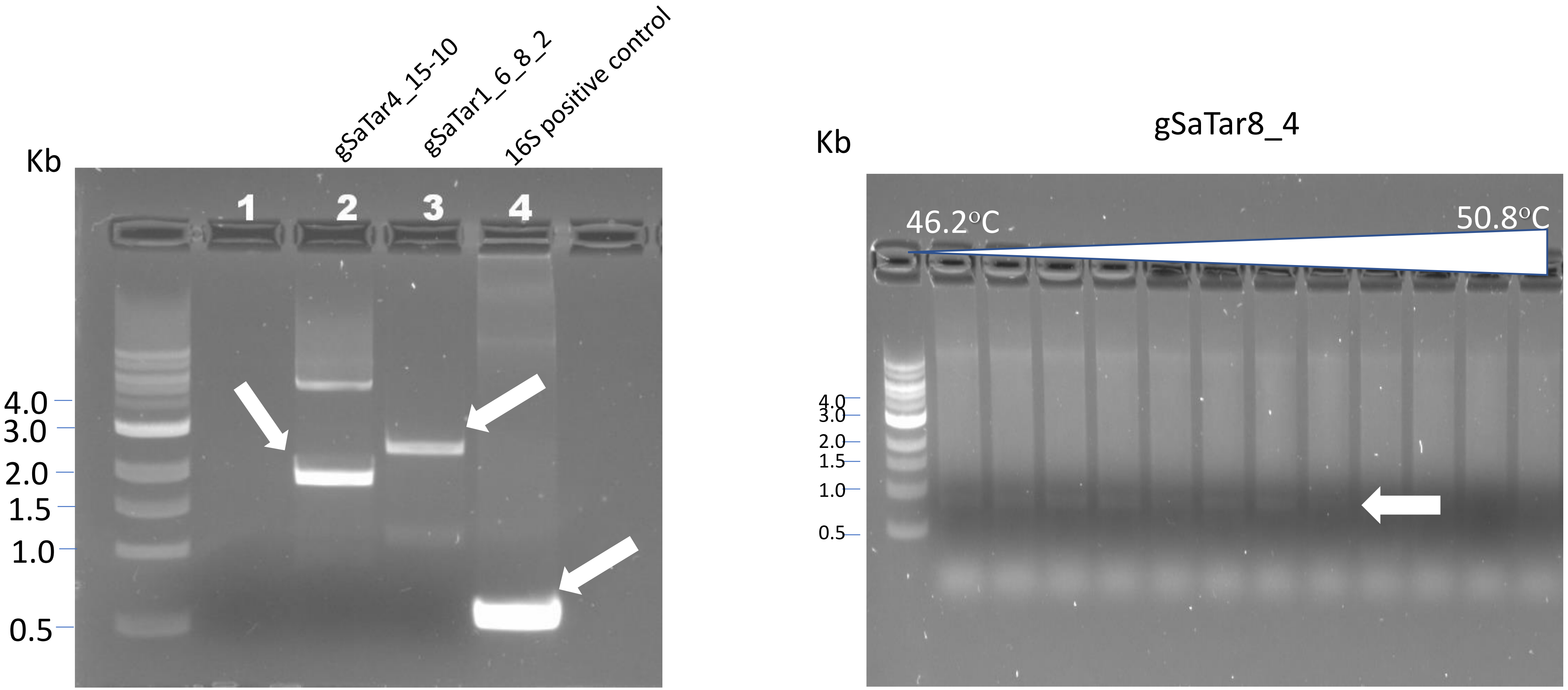

Additional file 17: PCR amplification of genomic regions containing gSaTar clusters.
PCR primers were designed in the flanking sequences of genomic regions containing gSaTar clusters. The structure feature of three genomic regions containing different number of gSaTar elements and their sequence information are provided in Additional file 3. 18S sequence was used as a positive control. The expected PCR products were indicated with arrows. Size for the DNA 1 kb ladder was provided. Note: PCR amplification of the genomic region containing SaTar4 and SaTar 8 cluster (gSaTar8_4) was performed under a temperature gradient condition as indicated.
