# Supplementary material for: Modular assembly of transposable element arrays by microsatellite targeting in the guayule and rice genomes
Source: BMC Genomics. 2018 Apr 19;19:271. doi: 10.1186/s12864-018-4653-6 (PMC5907723; doi:10.1186/s12864-018-4653-6)
Supplement: Supplementary file 11 — Alignment of rSaTar element conserved on Chromosome 1 of rice Japonica and Indica. (PDF 89 kb) [file 12864_2018_4653_MOESM11_ESM.pdf]

```
Japonica TTGGGGTCTAGGCACTCCCCTTAGCCAGTTACCAAAATTATCTTTTGCATTGCACCAAACAAAATTATCTTTTCCATTGTGGGCGCTCCCCTTAGCCAGCGATGTTTGATTAATTAAATTTTAAT
Indica   TTGGGGTCTAGGCACTCCCCTTAGCCAGTTACCAAAATTATCTTTTGCATTGCACCAAACAAAATTATCTTTTCCATTGTGGGCGCTCCCCTTAGCCAGCGATGTTTGATTAATTAAATTTTAAT

Japonica TATTTTATACTCATGAAATGGATTTATCTGATGTTACGTTTTCGCGTGATGCACCATTTAAAATGCGTAACTTATATATATATATATATATA-----TTTGTTTGGTGCTCCATGGTGCCCGGGC
Indica   TATTTTATACTCATGAAATGGATTTATCTGATGTTACGTTTTCGCGTGATGCACCATTTAAAATGCGTAACTTATATATATATATATATATAGTAAATTTGTTTGGTGCTCCATGGTGCCCGGGC

Japonica ACCATTGTTGAAATTGAGTATATACCCAATTCAAATGAGTATGAAACTTTTTCAATTACCTAGGTATTAACATTAACTACTTTACTAACTAGTTTAAAGCAAGTTTGAACTGAATTTGAACTTGT
Indica   AACATTGTTGAAATTGAGTATATACCCAATTCAAATGAGTATGAAACTTTTTCAATTACCTAGGTATTAACATTAACTACTTTACTAACTAGTTTAAAGCAAGTTTGAACTGAATTTGAACTTGT

Japonica TTTTGTTAGATTTTGATTCTAGGTATATAGCATCCATACATATAATTAGTTAGGTATGTAATCATGGATTGTGAAATAAAGTTAAATTTGAGTTGTTTTGTTGATAAGTATAGGTATGAATCAAA
Indica   TTTTGTTAGATTTTGATTCTAGGTATATAGCATCCATACATATAATTAGTTAGGTATGTAATCATGGATTGTGAAATAAAGTTAAATTTGAGTTGTTTTGTTGATAAGTATAGGTATGAATCAAA

Japonica TTATTATAACTAGGTATATACTCACAATTTGAAAATTTTGAAAAATTTGAGTGATTTTGTGCATTAGATACCATGGTGCCCGGGCACCATGGTGCCCCATATGAGTGTCCTATATATATATATAT
Indica   TTATTATAACTAGGTATATACTCACAATTTGAAAATTTTAAAAAATTTGAGTGATTTTGTGCATTAGATACCATGGTGCCCGGGCACCATGGTGCCCCATATGAGTGTCC--------------T

Japonica ATATATATATATATATATATATATATATATATATATATATATATAAAGTTTTTGCATGATGCACAATTTAAAATACGTGCTAACAAAAACTGAGACTGTATTAGAAGAAAGAAAAAGACCT-TAG
Indica   ATATATATATATATATATATATATATATATATATATATATATAAAAAGTTTTCGCATGATGCACAATTTAAAATACGTGCTAACAAAAACTGAGACTGTATTAGAAGAAAGAAAAAGACCTTTAG

Japonica TCTCACTCGCGAGACGCTACAAATATATAGTCCCCTCCACCGGCACTCACATCAACCAAAGCTGAAGAGCACACCACACCTTGTTAGTGGTGCACGATCGAGAGAGAAAGATCGATGAGAAGGT
Indica   TCTCACTCGCGAGACGCTACAAATATATAGTCCCCTCCACCGGCACTCACATCAACCAAAGCTGAAGAGCACACCACACCTTGTTAGTGGTGCACGATCGAGAGAGAAAGATCGATGAGAAGGT
```

rSaTar2

Additional file 11.

Alignment of rSaTar2 element conserved on chromosme 1 of rice Japonica and Indica. Japonica sequence from Oryza sativa (japonica) v7_JGI 42455894 - 42456770. Indica from Oryza sativa (indica) assembly ASM465v1 46535266 - 46536133.
